# Distinct mtDNA lineages in free‐ranging *Ammotragus*(aoudad) from the United States indicate multiple introductions from northern Africa

**DOI:** 10.1002/ece3.8849

**Published:** 2022-04-19

**Authors:** Emily A. Wright, Rachael C. Wiedmeier, Emma K. Roberts, David R. Pipkin, Froylán Hernández, Joseph P. Bayouth, Warren C. Conway, Robert D. Bradley

**Affiliations:** ^1^ 6177 Department of Biological Sciences Texas Tech University Lubbock Texas USA; ^2^ 6177 Climate Sciences Center Texas Tech University Lubbock Texas USA; ^3^ U.S. Department of Agriculture APHIS, Wildlife Services Canyon Texas USA; ^4^ 114669 Texas Parks and Wildlife Department Alpine Texas USA; ^5^ 6177 Department of Natural Resources Management Texas Tech University Lubbock Texas USA; ^6^ Museum of Texas Tech University Lubbock Texas USA

**Keywords:** *Ammotragus lervia*, cytochrome *b*, displacement loop, exotic species, *Ovis canadensis*, prion protein

## Abstract

Translocation records indicate aoudad (*Ammotragus lervia*) populations in the United States are a product of multiple human‐mediated introductions. Two mitochondrial markers (cytochrome *b*, cytb; displacement loop, D loop) and one nuclear gene (prion protein gene exon 3, *PRNP*) were used to determine: (1) genetic variation, (2) if genetic units correspond to taxonomic designations, (3) the number and geographic origin of translocations, and (4) divergence times. Three phylogenetic analyses (Bayesian inference, maximum likelihood, and parsimony) produced similar topologies with two clades (I and II). Clade I contained progeny of individuals resulting from introductions to Texas and Spain, and individuals from Algeria. Individuals in Clade II were progeny of past introductions to the United States and Europe, and northern Algeria. Clade II was subdivided into two subclades (A and B) representing two haplogroups. No genetic variation was detected in the *PRNP* sequences. Three haplogroups appeared to correspond to the subspecies *A. l. lervia* and *A. l. sahariensis* whose native distribution includes northwestern Africa. Network analyses assigned haplogroups to two major groups similar to those depicted in the phylogenetic analyses. Genetic distances ranged from 0.80% to 5.17% and 2.99% to 15.42% for cytb and D loop, respectively; and were higher than normally recovered for caprids, warranting a reexamination of subspecific status. Divergence dates indicated a major split between *A. l. lervia* and *A. l. sahariensis* circa 2.38 mya. Together, the high level of genetic divergences among US populations and apparent presence of two subspecies of aoudad in the United States support the hypothesis of multiple introductions from multiple sources.

## Graphical abstract

This manuscript explains that human‐mediated translocations of aoudad into the United States were a result of multiple introductions with putative subspecies identification as *A. l. sahariensis* and/or *A. l. blainei* and *A. l. lervia*. Two major clades were identified using phylogenetic, network, and divergence analyses, in which genetic distances ranged from 0.80% to 5.17% and from 2.99% to 15.42% for cytochrome *b* and displacement loop. Given the extensive number of free‐ranging aoudad currently in Texas, the manuscript provides baseline genetic data for future management and conservation plans being developed for a species that is considered “vulnerable” in its native range.

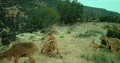

## 1 INTRODUCTION

**FIGURE 1:**
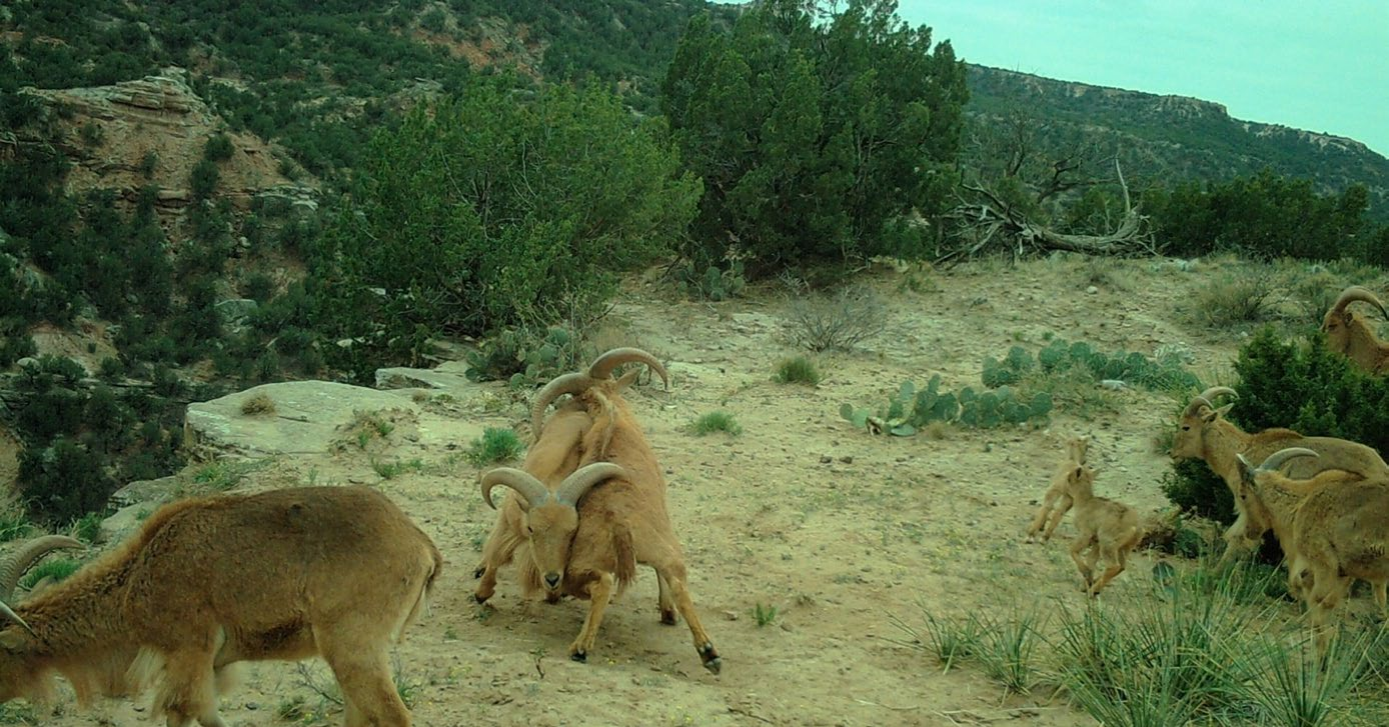
Photograph of *Ammotragus lervia* in Palo Duro Canyon, Texas

**FIGURE 2:**
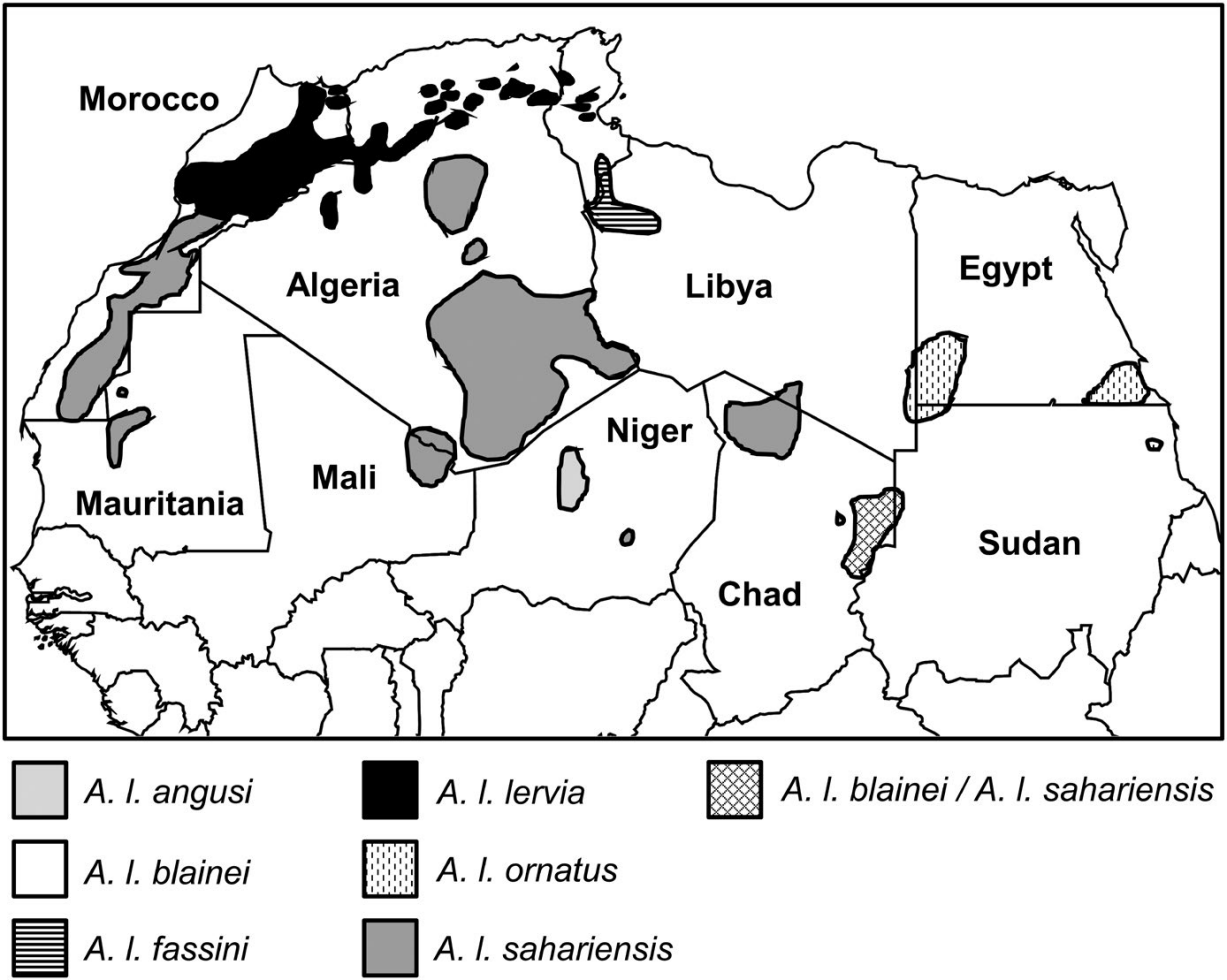
Map depicting the distribution of aoudad in North Africa based on Cassinello et al. (2008) and Cassinello (2013). Populations from northeastern Chad have been assigned to *A. l. blainei* (Alados et al., 1988) and *A. l. sahariensis* (Cassinello, 2013), in which resolving this is beyond the scope of this study. Therefore, this population is indicated by cross hashing to reflect its uncertainty

Aoudad, also known as barbary sheep or arrui (*Ammotragus lervia*, Pallas, 1777; see Figure 1), are native to the montane or massif regions of North Africa including Algeria, Chad, Egypt, Libya, Mali, Mauritania, Morocco, Niger, Sudan, and Tunisia (Cassinello et al., 2008). Based on morphology and geographical distribution, either four (Ellerman & Morrison‐Scott, 1951) or six subspecies of aoudad (Allen, 1939; Ansell, 1972; Cassinello, 1998; Gray & Simpson, 1980; Grubb, 2005; Harper, 1945) have been described. Cassinello et al. (2008) and Cassinello (2013) provide the most recent distributional information (see Figure 2) and taxonomic synopsis as follows: *A. l. lervia* (Atlas Aoudad), *A. l. ornatus* (Egyptian Aoudad), *A. l. blainei* (Kordofan Aoudad), *A. l. fassini* (Libyan Aoudad), *A. l. angusi* (Aïr Aoudad), and *A. l. sahariensis* (Saharan Aoudad). Little is known concerning genetic variation and phylogeographic patterns of diversity among these putative subspecies. In reviewing the taxonomy of Caprini, Groves and Grubb (2011) questioned whether *A. lervia* was a single species. The sole genetic study (Derouiche et al., 2020) included wild‐caught individuals from the Algerian provinces of Béchar, Illizi, and Tamanrasset; as well as semi‐captive individuals obtained from several preserves and zoos in Algeria. Their results based on mitochondrial DNA sequences, reflected a Mediterranean and Saharan divergence which seems to correspond to the two subspecies (*A. l. sahariensis* and *A. l. lervia*). To date, no genetic information is available for the other four subspecies, complicating the resolution of phylogenetic relationships among subspecies within their native range, as well as their role in determining source‐stock origins of introduced populations throughout the world.

A recent study (Stipoljev et al., 2021) used a mitochondrial marker (displacement loop, D loop) and microsatellite loci to determine genetic diversity and population structure in introduced populations of aoudad in Croatia, Czech Republic, and Spain. They reported evidence of four haplotypes and based on nuclear data they identified significant structure among populations. Stipoljev et al. (2021) suggested that the Almería haplotype probably was associated with *A. l. sahariensis*, whereas the other three haplotypes were of admixed origin presumably assignable to *A. l. lervia*. Stipoljev et al. (2021) reported low genetic diversity among populations (low number of detectable alleles and high number of shared alleles), consistent with a history of recent introductions (<50 years) and a small number of founding individuals from documented translocations.

**FIGURE 3:**
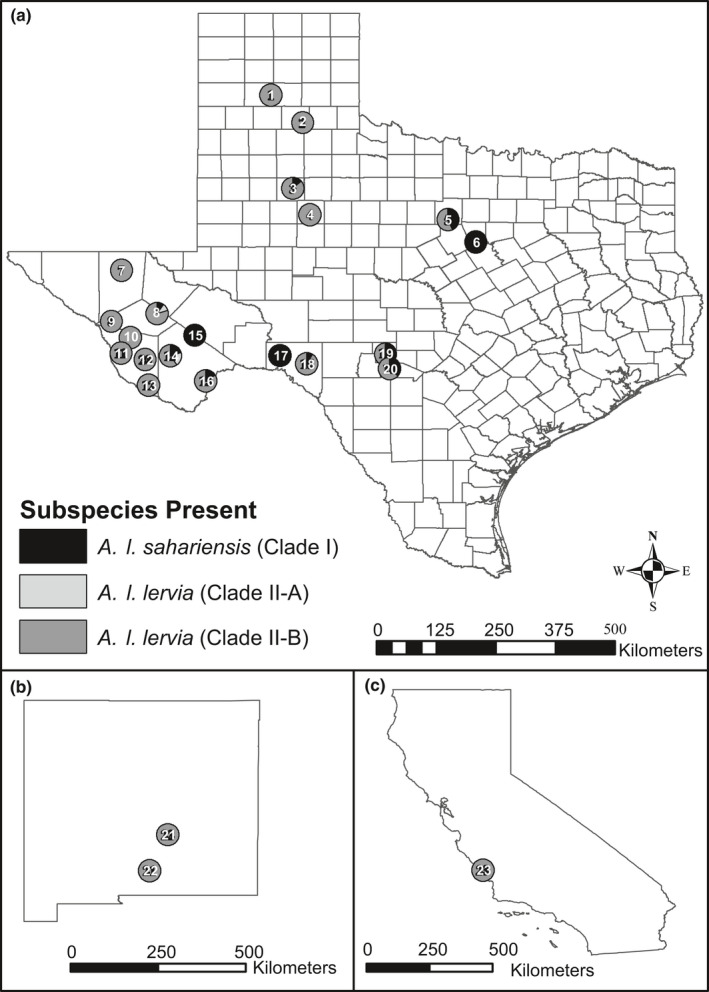
Map depicting sampling localities across Texas (3a), unless otherwise indicated: (1) Palo Duro Canyon State Park, (2) Caprock Canyons State Park, (3) Garza Co., (4) Scurry Co., (5) Fawcett Wildlife Management Area, (6) Fossil Rim Wildlife Center, (7) Culberson Co., (8) Davis Mountains (Jeff Davis and Reeves Counties), (9) Carrizo Mountains, (10) Van Horn Mountains, (11) Sierra Viejas Mountains, (12) Presidio County, (13) Chinati Mountains, (14) Elephant Mountain WMA, (15) Glass Mountains, (16) Black Gap WMA, 17) Val Verde County, (18) Dolan Falls Preserve, (19) Kerr WMA, (20) Love Creek Preserve, (21) Lincoln Co. (GMU 37), New Mexico (3b), (22) Alamogordo, New Mexico (3b), and (23) San Simeon, California (3c). Circles shaded black indicate localities with individuals represented in Clade I. Circles shaded in light gray indicate localities with individuals associated with Subclade II‐A. Circles shaded in dark gray represent localities where only individuals of Subclade II‐B were detected

Although aoudad are listed as “vulnerable” in their native range by the IUCN Red List of Threatened Species (Cassinello et al., 2008), substantial populations have been established in Europe and the southwestern United States (California, New Mexico, and Texas). In fact, the number of non‐native aoudad in the United States are thought to outnumber those existing in the native range (Cassinello et al., 2008; Stipoljev et al., 2021). Based on zoo records, it appears that aoudad initially were imported into the New York Zoological Park and the National Zoological Park in the United States, circa 1900 (Mungall & Sheffield, 1994; Ogren, 1959). Later, private ranches (William Randolph Hearst Ranch in California circa 1930 and Joe McKnight Ranch in New Mexico circa 1940) obtained progeny from various zoos across the United States for viewing and hunting opportunities, which are commonly thought to be the source of free‐ranging populations established in California and New Mexico (Barrett, 1980; Mungall & Sheffield, 1994; Ogren, 1965). In the 1950s, state agencies (New Mexico Department of Game and Fish, NMDGF and Texas Parks and Wildlife Department, TPWD) translocated aoudad from the Hearst and McKnight ranches into northeastern New Mexico and the Panhandle of Texas, respectively (DeArment, 1971, Mungall & Sheffield, 1994; Ogren, 1965). Further, throughout the 1950s and 1970s, private ranches independently introduced aoudad (Simpson & Krysl, 1981) into the eastern, central, and southwestern portions of Texas (Mungall & Sheffield, 1994). It is unclear whether these translocations included individuals previously established in Texas or were products of additional importations from their native range, zoos, or introduced populations in Europe. At present, similar translocation efforts and population expansion continue with aoudad now being common throughout the western two‐thirds of Texas. Currently, >30,000 free‐ranging aoudad are estimated to occur in Texas; with most populations residing in the Trans‐Pecos region, followed by the Edwards Plateau and Panhandle regions (F. Hernández, TPWD, personal communication; Traweek & Welch 1992; Figure 3); although aoudad occur in other ecoregions as a result of private introductions in high‐fenced, non‐free‐ranging operations, and subsequent escapees (Schmidly & Bradley, 2016).

Beyond the aforementioned zoo records and discussions presented in the *Symposium on Ecology and Management of Barbary Sheep* (Simpson, 1980) and *New Mexico Game and Fish Bulletins* (Ogren, 1962, 1965), there is little information relative to source‐stock origins and introductions into the United States and phylogenetic relationships of the six‐native subspecies in northern Africa. Most experts (Gray, 1985; Ogren, 1965) surmised that *A. l. lervia* served as the source‐stock for North American zoo introductions. Although most zoos did not retain detailed source of origin records (geographic history) in the early 1900s, those that documented the movement/transfers of aoudad between zoos (e.g., trading between the National Zoological Park and New York Zoological Park) were not always in agreement. Further, given that private ranches were not obligated to maintain source‐stock information or geographic history records, they generally did not provide point of origin data for imported aoudad. Consequently, the multiple imports and subsequent purchase or trading of zoo progeny by private individuals, coupled with a paucity of source/origin documentation, make it difficult to discern if more than one subspecies of aoudad was introduced into the United States during this time frame.

The introduction of exotic species into new geographical areas through human‐mediated translocations often inflict several direct and indirect ecological impacts on native species (Strauss et al., 2006). Of concern in the United States is the fact that aoudad are sympatric with native bighorn sheep (*Ovis canadensis*) (Simpson & Krysl, 1981). The potential for disease transmission and associated risks (e.g., epizootic hemorrhagic fevers, bluetongue, pneumonia, scrapie, and others; Candela et al., 2009; Cassmann et al., 2021; Fox et al., 2021; Hampy et al., 1979; Morawski et al., 2013; Richomme et al., 2006) and competition between aoudad and bighorn sheep (Barrett, 1967; McCarty & Bailey, 1994; Seegmiller & Simpson, 1979; Simpson et al., 1978) remains a high‐priority management concern.

Herein, we aim to provide the first broad‐scale geographic examination of aoudad in the southwestern United States (California, New Mexico, and Texas). The goals of this study were to: (1) assess genetic variation in free‐ranging populations of aoudad in Texas, California, and New Mexico, (2) determine if genetic units correspond to existing taxonomic designations, (3) ascertain the number and geographic source of introductions, and (4) provide estimates of approximate divergence times among haplogroups. Mitochondrial markers, cytochrome *b* (cytb), and displacement loop (D loop) and one nuclear gene (prion protein gene exon 3, *PRNP*) were used to determine the magnitude of genetic variation. The cytb marker is used widely as a proxy to measure genetic divergence among species and subspecies (Baker & Bradley, 2006; Bradley & Baker, 2001; Larsen et al., 2010). The D loop marker was selected because its rapid rate of nucleotide sequence evolution makes it ideal for examining genetic variation between and within populations and as a measure of nucleotide and haplotype diversity and other genetic indices (Latch et al., 2009; Mendez‐Harclerode et al., 2007; Stipoljev et al., 2021). DNA sequences for *PRNP* were available from other ongoing studies on aoudad and bighorn sheep; consequently, it was included as a means for detecting genetic variation in the nuclear genome.

## 2 MATERIALS AND METHODS

### 2.1 Sampling

A total of 232 aoudad samples were collected between 2018 and 2021, these included 209 samples from free‐ranging individuals, 19 samples from pedigreed captive individuals from the Fossil Rim Wildlife Center (Texas), and four historic samples (three from Palo Pinto County, Texas circa 1985 and one from Garza County, Texas circa 1991), and were used in this study (see Figure 3; Appendix). Samples (ear clip, muscle, liver, whole blood, and/or dried muscle) obtained in the United States were acquired through five methods: (1) hunter‐harvests facilitated by public hunts on Wildlife Management Areas (WMA), Game Management Units, State Parks, and private lands; (2) live‐captures in collaboration with TPWD and private landowners; (3) targeted removals in collaboration with TPWD and private landowners; (4) routine animal husbandry from the Fossil Rim Wildlife Center; and 5) destructive tissue loans borrowed from genetic resource collections housed in natural history museums: Natural Science Research Laboratory, Museum of Texas Tech University (NSRL) and Angelo State University Natural History Collection. Tissue samples obtained were either: (1) stored on ice and eventually frozen at −20°C or (2) immediately flash‐frozen in liquid nitrogen. All tissue samples and museum specimens were deposited into the NSRL. Specimens collected in the above procedures followed methods outlined in the guidelines of the American Society of Mammalogists (Sikes et al., 2016) and protocols approved by the Texas Tech University Animal Care and Use Committee (protocols #17023‐02 and 20002‐01).

### 2.2 DNA sequencing

Genomic DNA (gDNA) was extracted from 0.1 g ear clip, muscle, liver, or 100 µl blood (stored in standard collection tubes containing EDTA) using the Qiagen DNeasy blood and tissue extraction kit (Qiagen, Valencia, California). The entire cytb gene (1,143 bp) was amplified using the polymerase chain reaction (PCR) method (Saiki et al., 1988) with primers LGL765 (forward, Bickham et al., 1995) and LGL766 (reverse, Bickham et al., 2004), following the standard HotStarTaq (Qiagen Inc.) protocol: 25 µL reactions containing 30 ng of gDNA, 12.5 µl HotStarTaq premix, 8.3 µl of double‐distilled water, and 0.6 µl of each 10 µM primer. The thermal profile for PCR was as follows: hot start at 80°C, initial denaturation at 95°C for 2 min, followed by 34 cycles of denaturation at 95°C for 30 s, annealing at a range of 44–45°C for 45 s, and extension at 73°C for 1 min, with a final extension at 73°C for 15 min.

Polymerase chain reaction products were purified with ExoSAP‐IT PCR Product Cleanup (Applied Biosystems). Cycle sequencing reactions were conducted with BigDye Terminator v3.1 (Applied Biosystems) using the following primers: LGL766 and LGL765 (Bickham et al., 1995, 2004), 870R (Peppers et al., 2002), and F1 (Whiting et al., 2003). Cycle sequencing products subsequently were purified using Sephadex filtration (GE Healthcare) and centrifugation methods, followed by dehydration. Purified sequencing products were analyzed on an ABI 3730xl automated sequencer (Eurofins Genomics LLC). Resulting sequences were proofed using Sequencher 4.10.1 software (Gene Codes Corporation), and chromatograms generated from raw sequence reads were visually examined to authenticate all base changes. Verified sequences subsequently were aligned using MUSCLE version 3.5 (Edgar, 2004) for downstream analyses.

The mtDNA displacement loop (D loop) was amplified in select individuals based on resulting haplogroups identified from the cytb dataset. The differences in sequencing methods for D loop are described below. Primers utilized to amplify the full‐length D loop (1097 bp) were 2340‐4 (forward, Bickham et al., 1995) and 2340‐5 (reverse, Castro‐Campillo et al., 1999). Thermal profiles for PCR were as follows: a hot start of 80°C, initial denaturation at 95°C for 2 min, 95°C for 1 sec, 95°C for 1 min, 50°C for 1 s, annealing at a range of 48‐‐49°C for 1 min, extension at 72°C for 1 sec, followed by 35 cycles of denaturation at 95°C for 30 s, and a final extension at 72°C for 15 min. Primers used to cycle sequence the products included 2340‐4 (Bickham et al., 1995), 2340‐5 (Castro‐Campillo et al., 1999), 500F (Méndez‐Harclerode et al., 2005), and 1115 (Méndez‐Harclerode et al., 2005).

Data obtained from the cytb and D loop datasets were used to direct selection of individuals to be examined for the prion protein exon 3 gene (*PRNP*). Primers used to amplify the complete *PRNP* gene (771 bp) were MD582F (forward, Jewell et al., 2005) and MD1479RC (reverse, Jewell et al., 2005). Thermal profiles for PCR were as follows: a hot start of 80°C, initial denaturation at 95°C for 2 min, followed by 35 cycles of denaturation at 95°C for 30 s, annealing at 54°C for 45 s, and extension at 72°C for 1 min, with a final extension at 72°C for 15 min. Primers used to cycle sequence the products included MD582F, MD1479RC, 12, and 3FL1 (Jewell et al., 2005). All cytb, D loop, and *PRNP* sequences obtained in this study were deposited in GenBank (accession numbers: MZ507707‐MZ507938).

Additional sequence data for both mitochondrial markers (cytb: *n* = 17, D loop: *n* = 3) and the nuclear gene (*PRNP*: *n* = 1) datasets were obtained from NCBI GenBank and included samples examined in Derouiche et al. (2020), Stipoljev et al. (2021), as well as from unpublished studies. Inclusion of these samples broadened the sampling scheme to include individuals from the native range of aoudad as well as samples with a documented history (captive, semi‐captive, introduced, and zoo animals). Derouiche et al. (2020) summarized descriptions of localities from the literature that corresponded to GenBank accession numbers for sequences of cytb (in some cases, D loop as well for mitochondrial genomes) and noted several discrepancies in the reports describing the origin of non‐native individuals. However, Derouiche et al. (2020) and Stipoljev et al. (2021) provided exact localities for the cytb sequences for native aoudad in Algeria and D loop sequences for introduced aoudad into Europe, respectively.

### 2.3 Data analyses

#### 2.3.1 Phylogenetic analyses

In the following analyses, data were obtained from three independent studies: cytb only (Derouiche et al., 2020), D loop only (Stipoljev et al., 2021), and cytb and D loop combined (this study). A neighbor‐joining analysis (PAUP* version 4.0a169, Swofford, 2003) was conducted on 249 (232 sampled herein and 17 acquired from GenBank) individuals from the cytb dataset. The Arabian tahr (*Arabitragus jayakari* = *Hemitragus* *jayakari* by some authorities) was designated as the outgroup species following Ropiquet and Hassanin (2005) and Yang et al. (2013), to identify haplogroups and assignment of individuals to a clade. RAxML (version 8.2.12, Stamatakis, 2014) was used to detect and remove identical sequences, resulting in a final dataset of 23 haplotypes (unique sequences). This final dataset, containing 23 sequences, was used for all subsequent phylogenetic, genetic distance, and other genetic indices.

A parsimony analysis (PAUP* version 4.0a169, Swofford, 2003) was conducted to identify synapomorphies indicative of taxonomic identifications. Parsimony characters were assigned equal weight and variable nucleotide positions were treated as unordered, discrete characters with four possible states: A, C, G, and T. Phylogenetically uninformative characters were removed from the analysis. The most‐parsimonious trees were estimated using the heuristic search and tree‐bisection‐reconnection option. A strict consensus tree was generated from the population of most‐parsimonious trees and a subsequent bootstrap analysis (Felsenstein, 1985) with 1,000 iterations and the “fast” step‐wise option selected to evaluate nodal support.

Eighty‐eight maximum likelihood (ML) models were evaluated using jModelTest‐2.1.10 (Darriba et al., 2012; Guindon & Gascuel, 2003). The Akaike information criterion with a correction for finite sample sizes (AICc, Burnham & Anderson, 2004; Hurvich & Tsai, 1989) identified the Hasegawa‐Kishino‐Yano model of nucleotide substitution (HKY, Hasegawa et al., 1985) and proportion of invariable sites model (HKY+I, ‐lnL = 2279.6006) as the most appropriate for the cytb dataset. A likelihood analysis was performed using RAxML (version 8.2.12, Stamatakis, 2014) and the following parameters: base frequencies (A = 0.3205, C = 0.3012, G = 0.1273, and T = 0.2510), and the GTR + I + Γ (general time reversible plus proportion of invariable sites plus gamma distribution model of nucleotide substitution). Nodal support was evaluated using the bootstrap method (1,000 iterations, Felsenstein, 1985), with bootstrap values (BS) ≥ 65 used to indicate moderate‐to‐strong nodal support.

A ML analysis under a Bayesian inference (BI) model (MrBayes v3.2.6, Ronquist et al., 2012) was conducted to generate posterior probability values (PPV). The GTR + I + Γ nucleotide substitution model and the following parameters were used: two independent runs with four Markov chains (one cold and three heated; MCMCMC), 10 million generations, and sample frequency of every 1,000 generations from the last nine million generated. A visual inspection of likelihood scores resulted in the first 1,000,000 trees being discarded (10% burn‐in) and a consensus tree (50% majority rule) constructed from the remaining trees. PPV ≥ 0.95 were used to designate nodal support (Huelsenbeck et al., 2002).

The above phylogenetic methodologies were applied to the D loop dataset, which included a subset of 63 individuals (denoted in Appendix) from the cytb dataset generated herein as well as seven sequences obtained from GenBank, totaling 70 individuals. The differences in phylogenetic methods for D loop are described below. RAxML (version 8.2.12, Stamatakis, 2014) subsequently was used to detect and remove identical sequences, resulting in a final dataset of 36 haplotypes (unique sequences). This final dataset was used for all subsequent phylogenetic, genetic distance, and other genetic indices. Eighty‐eight ML models were evaluated using jModelTest‐2.1.10 (Darriba et al., 2012; Guindon & Gascuel, 2003) and the AICc (Burnham & Anderson, 2004; Hurvich & Tsai, 1989) identified the Hasegawa‐Kishino‐Yano model of nucleotide substitution (HKY, Hasegawa et al., 1985) and gamma distribution (HKY+Γ, ‐lnL = 2985.7232) as the most appropriate for the D loop dataset. A likelihood analysis was performed using the following parameters: base frequencies (A = 0.3165, C = 0.2575, G = 0.1460, and T = 0.2801), and gamma distribution (G = 0.3170) and the GTR + I + Γ in the program RAxML (version 8.2.12, Stamatakis, 2014).

#### 2.3.2 Genetic divergence

Genetic distance values for selected taxa and mitochondrial haplogroups were estimated using the Kimura 2‐parameter model of evolution (Kimura, 1980) and the Tamura‐Nei model of evolution (Tamura & Nei, 1993) for the cytb and D loop datasets, respectively, using the program MEGA X (Kumar et al., 2018). The resulting values calculated from the mitochondrial markers were used to examine levels of genetic divergence pertaining to the genetic species concept outlined in Bradley and Baker (2001) and Baker and Bradley (2006). Sequences of both cytb and D loop for closely related taxa (Yang et al., 2013) were obtained from GenBank to provide comparative genetic distance values.

#### 2.3.3 Divergence dating

A Molecular Clock Test (ML, Kumar et al., 2018) was used to accept or reject a strict molecular clock. This result was used with the software program BEAST v2.6.2 (Bouckaert et al., 2019) as a prior to estimate molecular timelines associated with phylogenetic divergence. Divergence dates for aoudad were estimated from the reduced cytb dataset (as explained above) using *Rupicapra rupicapra*, *R. pyrenaica*, and *Arabitragus jayakari* as outgroup taxa. Fossil calibrations were placed on the *Rupicapra* node, based on a fossil date (~8.0 mya) obtained from an estimation of divergence from the most recent common ancestor (Derouiche et al., 2020; Hassanin et al., 2012; Wendorf & Schild, 1976) following methods outlined in previous studies (Ordóñez‐Garza et al., 2014; Thompson et al., 2015; Wright et al., 2021). A Yule tree prior was used in the BEAST analysis and a prior lognormal distribution was placed on root height to constrain the divergence date estimates of the overall tree to the estimated fossil date (~8.0 mya) with a σ value of 0.5 and to reflect the uncertainty of the fossil record. Optimization of the analysis and determination of final parameters were examined using initial test runs with the following parameters: GTR + I + Γ, 1 × 10^7^ generations, and 10% burn‐in. Initial test runs using the GTR + I + Γ model of nucleotide substitution yielded low values of effective sample size, necessitating the selection of a simpler model. Therefore, HKY + I + Γ model of nucleotide substitution was used to minimize the effects of over‐parameterization on effective sample size. A final run of 50 x 10^7^ generations was analyzed with log and tree files, which were then combined to generate divergence date estimates and produce a maximum clade credibility tree. The program Tracer (Rambaut et al., 2018) was used to examine number of successful MCMC iterations, stability of topological convergence, and Effective Sample Size using a value >200 as indicative of a minimal threshold for all parameters. The program TreeAnnotator (Bouckaert et al., 2019) subsequently was used to obtain an estimate of the final phylogenetic tree with divergence dates assigned to nodes.

#### 2.3.4 Diversity and haplotype analyses

The number of polymorphic sites (*s*), nucleotide diversity (π), number of haplotypes (*h*), haplotype diversity (*H*_d_), and Fu's test of neutrality were calculated for both the entire cytb and D loop dataset (excluding sites with gaps or missing data and sequences from individuals located outside of the United States) using DNAsp v6 (Rozas et al., 2017). The program Arlequin v3.5.2.2 (Excoffier & Lischer, 2010) was used to calculate the analysis of molecular variance (AMOVA‐Excoffier et al., 1992; Weir, 1996; Weir & Cockerham, 1984).

A median‐joining network analysis (Network 10.2.0.0 Fluxus Technology Ltd 2021, Bandelt et al., 1999) was conducted for both the cytb and D loop datasets to determine relationships between haplogroups. The program DNAsp v6 was used to remove invariable sites, ignore gaps/missing data, and determine haplotypes within the datasets.

#### 2.3.5 Characterization of PRNP

*PRNP*sequences were proofed using Sequencher 4.10.1 software (Gene Codes Corporation, Ann Arbor, Michigan) and heterozygous nucleotide base positions were visually determined using chromatograms. The program MEGA X (Kumar et al., 2018) was used to translate the nucleotide sequences to protein, allowing for the detection of any non‐synonymous substitutions based on an outgroup comparison to closely related genera (*Capra* and *Ovis*). In particular, three codons known to be of importance (Goldmann, 2008) in identifying susceptibility to scrapie in domestic sheep and goats were examined. These polymorphic codons included: A136V/T, R154H/L, and Q171R/H/K (referenced using traditional amino acid terminology, Dunnen & Antonarakis, 2000).

## 3 RESULTS

### 3.1 Phylogenetic analyses

#### 3.1.1 Cytochrome *b* dataset

**FIGURE 4:**
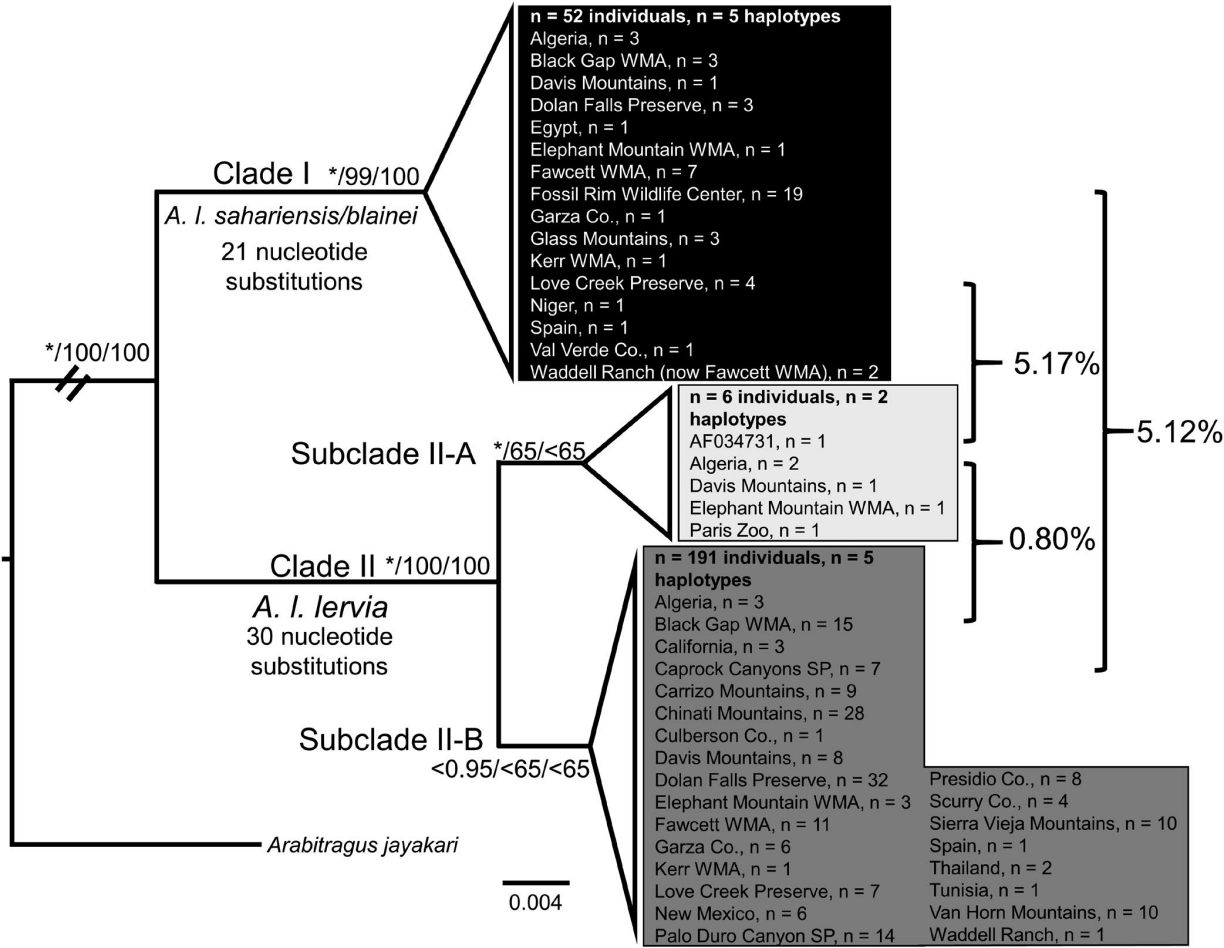
Phylogeny of the cytochrome *b* gene using all individuals. Bayesian posterior probability values are indicated by the * and represent ≥ 0.95 nodal support and likelihood bootstrap values are represented right of the slash where bootstrap values ≥ 65 indicate nodal support. Specific localities are located in Texas unless otherwise denoted

For the cytb dataset, the preliminary neighbor‐joining analysis (not shown) of 249 individuals was used to assign haplotype affiliation of all individuals to the sampled localities (see Figure 3). From this analysis, a reduced dataset (*n* = 23) was obtained when identical sequences were removed. This final dataset was used for all subsequent analyses involving phylogenetics, genetic distances, and genetic indices. The three phylogenetic analyses (BI, ML, and parsimony) generated similar topologies in the cytb dataset; consequently, each analysis is discussed in detail below; however, only the topology obtained from the BI analysis is shown (Figure 4). Although there was substantial variation among individuals in terminal nodes, these associations were collapsed due to lack of nodal support.

In the BI analysis, two supported clades were identified (I and II). Clade I contained 52 individuals from western and southeastern Algeria; Black Gap WMA, Davis Mountains, Dolan Falls Preserve, Elephant Mountain WMA, Fawcett WMA, Fossil Rim Wildlife Center, Garza Co., Glass Mountains, Kerr WMA, Love Creek Preserve, Val Verde Co., and the Waddell Ranch (now Fawcett WMA) in Texas; Mansoura Zoo, Egypt and/or Niger; and Navalvillar de Pela region, Spain. Clade II was divided into two subclades (A and B); with Subclade II‐A being supported in the BI analysis (PPV ≥ 0.95). The three haplotypes representing the 191 individual sequences forming Subclade II‐B were not supported by the three analyses and instead formed an unresolved polytomy. Subclade II‐A contained 6 individuals from the Béchar Province, Algeria; Elephant Mountain WMA and Davis Mountains in Texas; National Museum of Natural History (MNHN), Paris, France; and Vincennes Zoo, Paris, France and/or Morocco and/or La Hoya Field Station, Almería, Spain. Subclade II‐B contained 191 individuals from northern Algeria; northern Algeria and/or La Hoya Field Station and/or Tunisia, Almería, Spain; Black Gap WMA, Caprock Canyons State Park, Carrizo Mountains, Chinati Mountains, Culberson Co., Davis Mountains, Dolan Falls Preserve, Elephant Mountain WMA, Fawcett WMA, Garza Co., Kerr WMA, Love Creek Preserve, Palo Duro Canyon State Park, Presidio Co., Scurry Co., Sierra Vieja Mountains, Van Horn Mountains, and Waddell Ranch in Texas; Cambria, California; Game Management Units near Lincoln National Forest, New Mexico; Thailand; and Tunisia.

The ML analysis also produced a topology (not shown) that was nearly identical to the topology obtained from the BI analysis. The only difference between the BI and ML analyses involved nodal support for Subclade II‐A. In the ML analysis, Subclade II‐A was only moderately supported (BS = 65). Similar to the BI analysis, no support was obtained for the three haplotypes representing the 191 individual sequences forming Subclade II‐B and produced an unresolved polytomy. Bootstrap support values obtained from the ML analysis were superimposed onto the BI topology (Figure 4).

For the parsimony analysis, 379,908 equally, most parsimonious trees (length = 143, homoplasy index = 0.0909, and consistency index = 0.9091) were retrieved. A majority rule consensus tree was generated (not shown) that was similar in topology to the tree obtained in the BI analysis (Figure 4); consequently, the bootstrap support values from the parsimony analysis were superimposed onto the BI topology.

In the parsimony analysis (not shown), there was no nodal support (BS < 65) for either Subclade A or B resulting in an unresolved polytomy containing seven haplotypes representing the 197 individual sequences. Bootstrap support values obtained from the parsimony analysis were superimposed onto the BI topology (Figure 4). The 51 nucleotide substitutions that were determined to be phylogenetically informative were superimposed onto the topology obtained from the BI analysis, with 21 acting as synapomorphies for Clade I and 30 acting as synapomorphies for Clade II. Translation of these 51 nucleotide substitutions to amino acids resulted in the identification of five nonsynonymous substitutions that differentiated Clade I (L303I) from Clade II (L121F, T122A, M240T, I348M).

#### 3.1.2 D loop dataset

**FIGURE 5:**
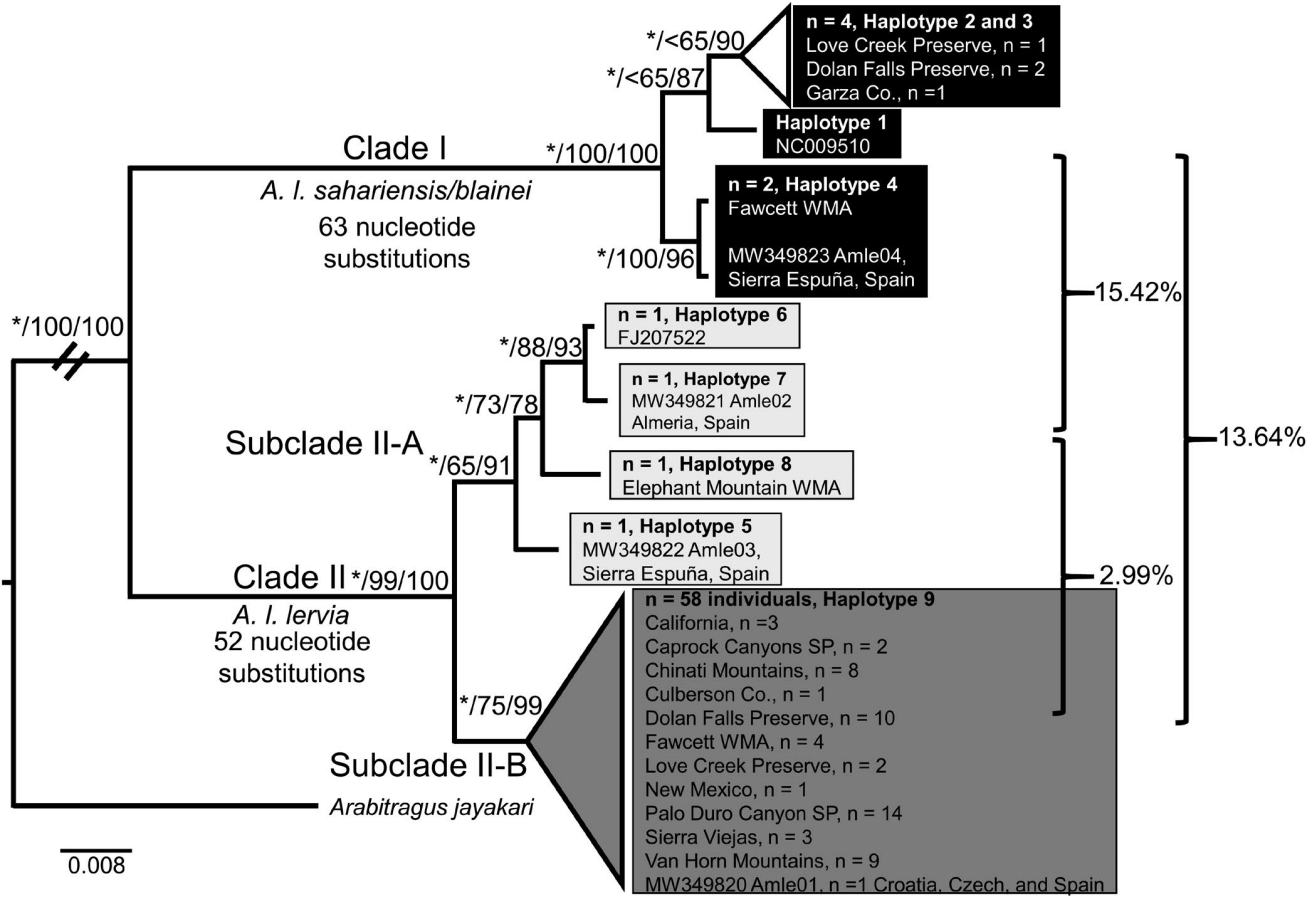
Phylogeny of D loop using selected individuals based on a priori results from the cytb gene. Bayesian posterior probability values are indicated by the * and represent ≥ 0.95 nodal support and likelihood bootstrap values are represented right of the slash where bootstrap values ≥ 65 indicate nodal support. Specific localities are located in Texas unless otherwise denoted

For the D loop dataset, the preliminary neighbor‐joining analysis (not shown) of 70 aoudad samples was used to assign haplotype affiliation of all individuals to the sampled localities (see Figure 3). From this analysis, a reduced dataset (*n* = 36) was obtained when identical sequences were removed. This final dataset then was used for all subsequent phylogenetic analyses, estimations of genetic distances, and other calculation of genetic indices. The three phylogenetic analyses (BI, ML, and parsimony) generated similar topologies; consequently, each analysis is discussed in detail below; however, only the topology obtained from the BI analysis is shown (Figure 5).

The BI analysis of the D loop dataset indicated two supported clades (I and II) of aoudad samples (Figure 5). Clade I contained sequences corresponding to individuals from Love Creek Preserve, Dolan Falls Preserve, Garza Co., Fawcett WMA; Sierra Espuña, Spain; and Mansoura Zoo, Egypt and/or Niger. Clade II was divided into two subclades (A and B) supported in all three analyses. Subclade II‐A contained individuals from Elephant Mountain WMA in Texas; Vincennes Zoo, Paris, France and/or Morocco and/or La Hoya Field Station, Almería, Spain; Almería, Spain; and Sierra Espuña, Spain. Subclade II‐B contained individuals represented by the following localities: Cambria, California; Game Management Units near Lincoln National Forest, New Mexico; Caprock Canyons State Park, Chinati Mountains, Dolan Falls Preserve, Palo Duro Canyon State Park, Sierra Viejas Mountains, Van Horn Mountains in Texas; Mosor Mountain, Croatia; region near Plzeň, Czech; and Sierra Espuña, Spain.

The ML analysis of the D loop dataset also produced a topology (not shown) that was essentially identical to the topology obtained from the BI analysis. As in the BI analysis, strong support was recovered for Clades I and II (BS = 100 and BS = 99, respectively) as was moderate support for Subclade II‐A and Subclade II‐B (BS = 65 and BS = 75, respectively). Bootstrap support values obtained from the ML analysis were superimposed onto the BI topology (Figure 5).

Due to computation limitations (tree storage issues) for the parsimony analysis of the D loop dataset, the heuristic search was terminated before the analysis could be completed. At the point of termination (total number of rearrangements tried = 355,813,677), 9,461,411 equally, most‐parsimonious trees (length = 289, homoplasy index = 0.1003, and consistency index = 0.8997) were retrieved. The parsimony analysis produced a topology (not shown) that was essentially identical to the topology of the BI analysis. Bootstrap support values obtained from the parsimony analysis were superimposed onto the BI topology (Figure 5). As in the BI analysis, strong support was recovered for Clades I and II (BS = 100 and BS = 100, respectively) as was strong support for Subclade II‐A and Subclade II‐B (BS = 91 and BS = 99, respectively). The 118 nucleotide substitutions that were determined to be phylogenetically informative were superimposed onto the topology obtained from the BI analysis, with 63 acting as synapomorphies for Clade I and 52 acting as synapomorphies for Clade II.

### 3.2 Insertion and deletion events

Ten insertion and deletions events (indels), representing a total of 64 nucleotide substitutions, were detected in the D loop dataset (Table 1). These indels occurred between nucleotides sites 15,556 to 15,772 (aoudad reference genome, NC009510). Deletions ranged from a single nucleotide to 16 bp, whereas insertions ranged from two to 15 nucleotides. Several of the indels were of phylogenetic relevance, for example, the first deletion event was 15 bp and restricted to individuals in Clade II, whereas the second deletion event was 16 bp in length and restricted to individuals in Clade I. Collectively, these indels contributed to the greater branch lengths depicted in the D loop topology relative to the cytb dataset.

**TABLE 1:** Multiple insertion and deletions events (indels) detected in the D loop dataset. These indel events occurred between sites 15,556–15,772 in the aoudad reference genome (NC009510). Shown is a 68 bp region out of the approximately 325 bp region

Animal	Identification	Sequence
*H. jayakari*	NC020621	‐‐‐‐‐‐‐‐‐‐‐‐‐‐‐CAAA‐‐‐‐‐‐‐‐‐‐‐‐‐‐‐‐‐‐‐‐‐CATGAAA‐‐‐‐‐‐‐TCAACACCATACAA‐TGCAAACG‐‐‐‐‐‐‐‐‐‐‐‐‐
*A. lervia*	NC009510	ACAATTTTCACTCACCAAACGCAGCACCCCATCACCC‐‐‐‐‐‐‐‐‐‐‐‐‐‐‐‐TTCAACCTAACCCAA‐CGCGGACG‐ATGCATGTGAAT
*A. lervia*	Clade I	ACAATTTTCACTCACCAAACGCAGCACCCCATCACCC‐‐‐‐‐‐‐‐‐‐‐‐‐‐‐‐TTCAACCTAACCCAAGCGCGGACGCATGCATGTGAAT
*A. lervia*	Clade II	‐‐‐‐‐‐‐‐‐‐‐‐‐‐‐CAAATACACTACACCACCCGTCCTACAAGAAATAGATATTCAACGCTATGCAA‐‐ACAAACACAC‐‐‐‐‐‐‐AGT

### 3.3 Genetic distances

Estimation of Kimura‐2 parameter (Kimura, 1980) genetic distances (Table 2), obtained from the cytb dataset, indicated that the average genetic distance among all individuals included in the study was 2.73%; whereas distances within selected clades were as follows: individuals comprising Clade I was 0.32%; 0.48% for individuals constituting Clade II; and 0.29% and 0.48% for Subclade II‐A and Subclade II‐B, respectively. Estimates for genetic distances between clades were: 0.80% between Subclade II‐A and Subclade II‐B; 5.12% between Clades I and Clade II; 5.17% between Clade I and Subclade II‐A; and 5.12% % between Clade I and Subclade II‐B (Figure 4).

**TABLE 2:** Average genetic distances of cytb sequences estimated using the Kimura 2‐parameter model of evolution (Kimura, 1980) for selected comparisons of aoudad and taxa of Family Bovidae

Comparison	Average Genetic Distance
Within subspecies	
*Ammotragus lervia*(Clade I)	0.32%
*Ammotragus lervia*(Clade II)	0.48%
*Ammotragus lervia*(Clade II‐A)	0.29%
*Ammotragus lervia*(Clade II‐B)	0.48%
*Budorcas taxicolor taxicolor*	0.49%
*B. t. tibetana*	0.49%
*B. t. bedfordi*	0.25%
*Rupicapra rupicapra rupicapra*	1.32%
*R. r. tatrica*	0.28%
*R. r. cartusiana*	2.64%
*R. r. carpatica*	0.29%
*R. pyrenaica pyrenaica*	0.30%
*R. p. ornata*	0.19%
*R. p. parva*	0.46%
Within species	
*A. lervia*	2.73%
*Hemitragus jemlahicus*	2.11%
*Oreamnos americanus*	1.91%
*Budorcas taxicolor*	2.43%
*Pseudois nayaur*	3.19%
*Pseudois schaeferi*	1.86%
*R. rupicapra*	1.72%
*R. pyrenaica*	1.49%
Between subspecies	
*A. lervia*(Clade I) – *A. lervia* (Clade II)	5.12%
*A. lervia*(Clade I) – *A. lervia* (Subclade II‐A)	5.17%
*A. lervia*(Clade I) – *A. lervia* (Subclade II‐B)	5.12%
*A. lervia*(Subclade II‐A) – *A. lervia* (Subclade II‐B)	0.80%
*B. t. taxicolor – B. t. tibetana*	3.51%
*B. t. taxicolor – B. t. bedfordi*	1.01%
*B. t. tibetana – B. t. bedfordi*	2.54%
*R. r. rupicapra – R. r. tatrica*	1.24%
*R. r. rupicapra – R. r. cartusiana*	3.99%
*R. r. rupicapra – R. r. balcanica*	1.01%
*R. r. rupicapra – R. r. carpatica*	1.41%
*R. r. rupicapra – R. r. caucasica*	2.14%
*R. r. rupicapra – R. r. asiatica*	1.30%
*R. r. tatrica – R. r. cartusiana*	4.05%
*R. r. tatrica – R. r. balcanica*	0.10%
*R. r. tatrica – R. r. carpatica*	0.53%
*R. r. tatrica*– *R. r. caucasica*	1.25%
*R. r. tatrica*– *R. r. asiatica*	0.38%
*R. r. cartusiana*– *R. r. balcanica*	3.10%
*R. r. cartusiana*– *R. r. carpatica*	2.95%
*R. r. cartusiana*– *R. r. caucasica*	3.70%
*R. r. cartusiana*– *R. r. asiatica*	3.41%
*R. r. balcanica*– *R. r. carpatica*	0.43%
*R. r. balcanica*– *R. r. caucasica*	1.16%
*R. r. balcanica*– *R. r. asiatica*	0.29%
*R. r. carpatica*– *R. r. caucasica*	0.72%
*R. r. carpatica*– *R. r. asiatica*	0.72%
*R. r. caucasica*– *R. r. asiatica*	1.45%
*R. p. pyrenaica*– *R. p. ornata*	3.15%
*R. p. pyrenaica*– *R. p. parva*	1.20%
*R. p. ornata*– *R. p. parva*	2.26%
Between species	
*H. hylocrius – H. jayakari*	7.89%
*H. hylocrius – H. jemlahicus*	10.15%
*H. jayakari – H. jemlahicus*	8.34%
*P. nayaur – Pseudois schaeferi*	2.92%
*R. rupicapra – R. pyrenaica*	4.14%

Genetic distances (see Table 3) obtained from the D loop dataset were estimated using the Tamura and Nei model of evolution (Tamura & Nei, 1993). The average genetic distance among all individuals included in the study was 4.57%; whereas distances within selected clades were as follows: individuals comprising Clade I was 1.28%; 0.84% for members of Clade II; and 1.48% and 0.14% Subclade II‐A and Subclade II‐B, respectively. Estimates for genetic distances between clades were: 2.99% between Subclade II‐A and Subclade II‐B; 13.88% between Clades I and II; 15.42% between Clade I and Subclade II‐A, and 13.64% between Clade I and Subclade II‐B (Figure 5).

**TABLE 3:** Average genetic distances of D‐loop sequences estimated using the Tamura‐Nei model of evolution (Tamura & Nei, 1993) for selected comparisons of aoudad and taxa of the Subfamily Caprinae

Comparison	Average Genetic Distance
Within subspecies	
*Ammotragus lervia*(Clade I)	1.28%
*Ammotragus lervia*(Clade II)	0.84%
*Ammotragus lervia*(Clade II‐A)	1.48%
*Ammotragus lervia*(Clade II‐B)	0.14%
*Rupicapra rupicapra rupicapra*	3.41%
*R. r. tatrica*	0.34%
*R. r. cartusiana*	0.32%
*R. r. balcanica*	4.01%
*R. r. carpatica*	2.51%
*R. r. caucasica*	2.31%
*R. pyrenaica pyrenaica*	2.02%
*R. p. ornata*	0.07%
*R. p. parva*	2.45%
Within species	
*A. lervia*	4.57%
*R. rupicapra*	4.18%
*R. pyrenaica*	5.45%
*Oreamnos americanus*	3.85%
*Budorcas taxicolor*	2.42%
*Pseudois nayaur*	14.18%
Between subspecies	
*A. lervia*(Clade I) – *A. lervia* (Clade II)	13.88%
*A. lervia*(Clade I) – *A. lervia* (Subclade II‐A)	15.42%
*A. lervia*(Clade I) – *A. lervia* (Subclade II‐B)	13.64%
*A. lervia*(Subclade II‐A) – *A. lervia* (Subclade III‐B)	2.99%
*R. r. rupicapra*– *R. r. tatrica*	3.76%
*R. r. rupicapra*– *R. r. cartusiana*	13.23%
*R. r. rupicapra*– *R. r. balcanica*	6.31%
*R. r. rupicapra*– *R. r. carpatica*	5.98%
*R. r. rupicapra*– *R. r. caucasica*	6.68%
*R. r. rupicapra*– *R. r. asiatica*	6.67%
*R. r. tatrica*– *R. r. cartusiana*	11.86%
*R. r. tatrica*– *R. r. balcanica*	5.44%
*R. r. tatrica*– *R. r. carpatica*	4.13%
*R. r. tatrica*– *R. r. caucasica*	5.38%
*R. r. tatrica*– *R. r. asiatica*	7.05%
*R. r. cartusiana*– *R. r. balcanica*	13.65%
*R. r. cartusiana*– *R. r. carpatica*	12.93%
*R. r. cartusiana*– *R. r. caucasica*	14.18%
*R. r. cartusiana*– *R. r. asiatica*	14.59%
*R. r. balcanica*– *R. r. carpatica*	6.35%
*R. r. balcanica*– *R. r. caucasica*	5.05%
*R. r. balcanica*– *R. r. asiatica*	5.81%
*R. r. carpatica*– *R. r. caucasica*	5.43%
*R. r. carpatica*– *R. r. asiatica*	6.35%
*R. r. caucasica*– *R. r. asiatica*	3.28%
*R. p. pyrenaica*– *R. p. ornata*	11.67%
*R. p. pyrenaica*– *R. p. parva*	3.82%
*R. p. ornata*– *R. p. parva*	13.29%
Between species	
*R. rupicapra – R. pyrenaica*	11.51%
*Pseudois nayaur*– *Pseudois schaeferi*	18.28%

### 3.4 Divergence dating

**FIGURE 6:**
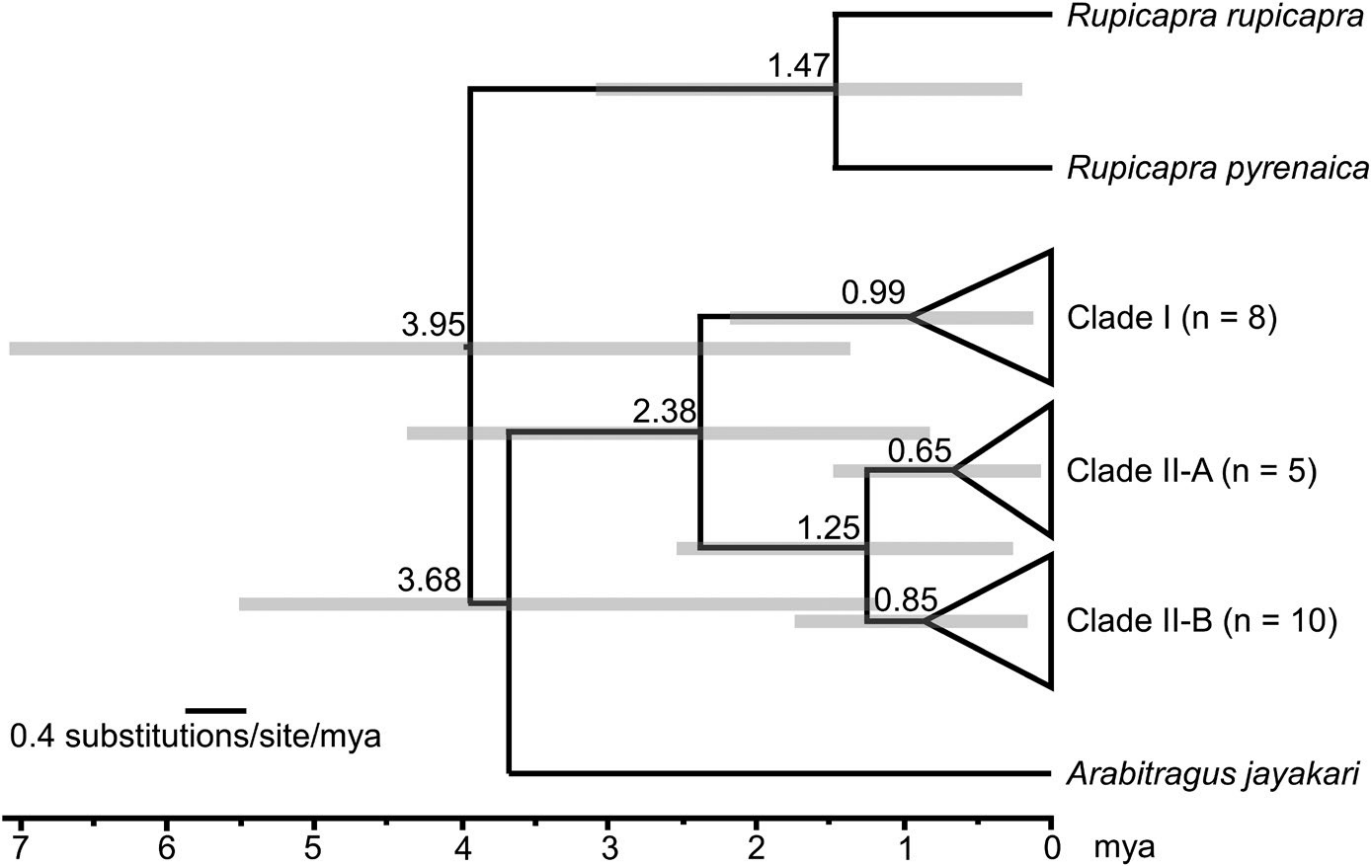
Time‐calibrated phylogenetic tree modified from that depicted in Figure 4^2^ with the superimposition of results from the BEAST analysis (version 2.6.1, Bouckaert et al., 2014) using the reduced mitochondrial cytochrome *b* gene dataset. Divergence date estimates are indicated along the x‐axis in millions of years. Error bars (gray rectangles) represent the 95% highest posterior density for node height

A Molecular Clock Test (Kumar et al., 2018) determined that the null hypothesis of equal rates of molecular evolution throughout the tree were indicative of a relaxed molecular clock. The BEAST analyses depicted a mean divergence rate of 0.0116 substitutions per site per million years (95% highest posterior density [HPD]: 0.0043–0.0211) for cytb (Figure 6). The Yule birth rate was estimated to be 1.2621 (95% HPD: 0.3809–2.3908). The divergence dating analysis indicated the initial divergence of Clade I (*A. l. sahariensis*) from Clade II (*A. l. lervia*) began approximately 2.38 mya. Radiation within *A. l. sahariensis* (Clade I) was estimated to have occurred at 0.99 mya. The major split between individuals assigned to Subclade II‐A and Subclade II‐B is estimated within the last 1.25 mya followed by radiation within Subclade II‐A and Subclade II‐B was estimated at 0.65 and 0.85 mya, respectively.

### 3.5 Diversity and haplotype analyses

**FIGURE 7:**
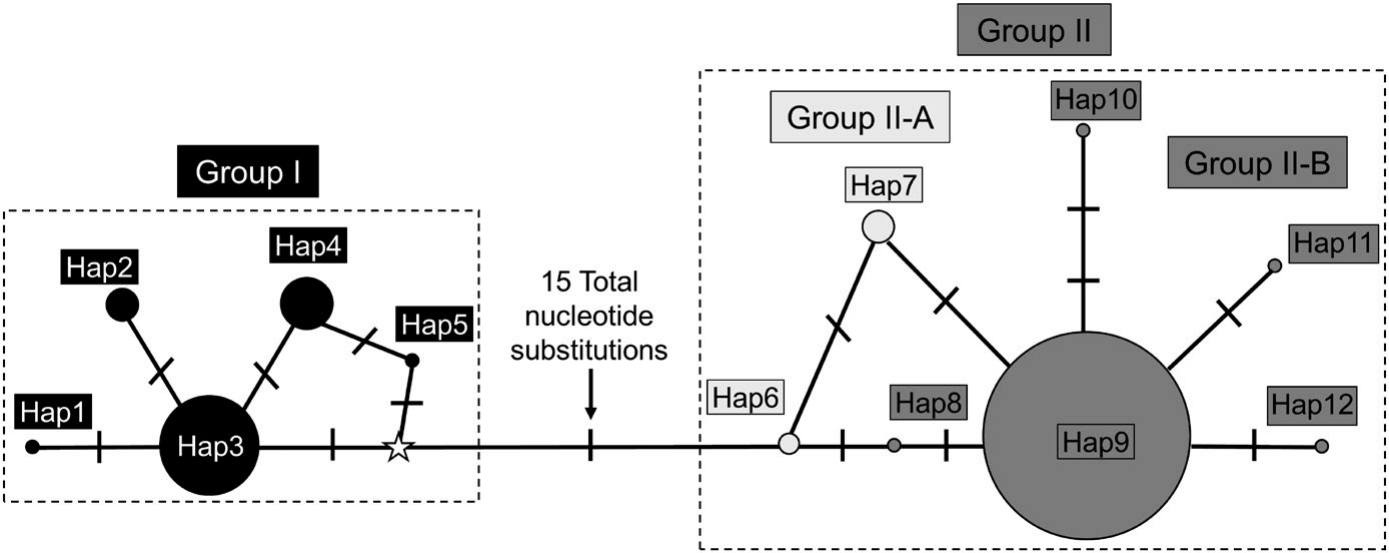
A median‐joining haplotype network of the entire cytochrome *b* dataset, including all sequences from the United State and GenBank. Haplotypes are represented by circles with sizes proportional to the number of associated individuals. Number of mutations between nodes is represented by slashes unless otherwise noted

**FIGURE 8:**
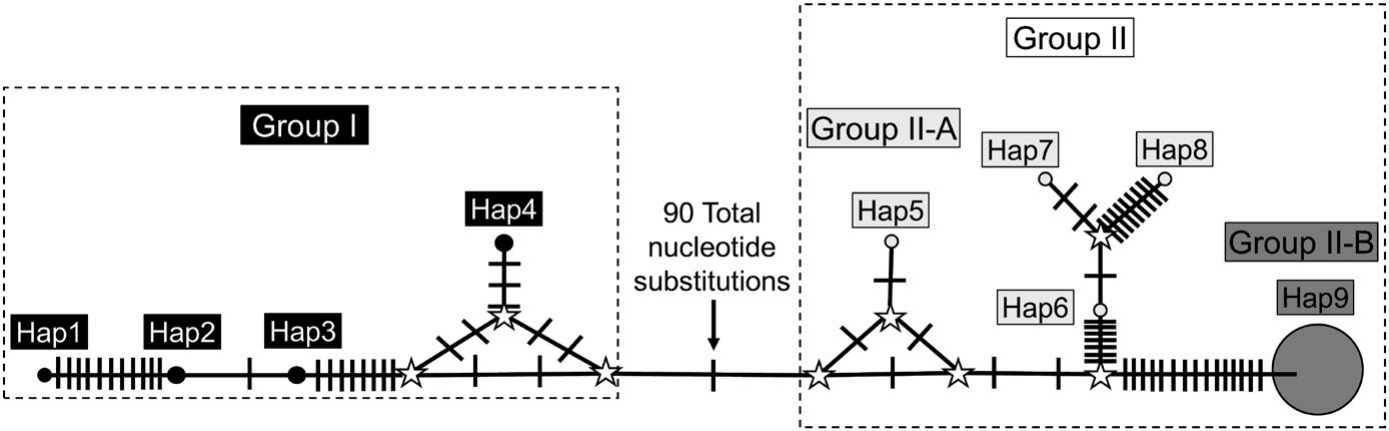
A median‐joining haplotype network of the entire D loop dataset, including all sequences from the United States and GenBank. Haplotypes are represented by circles with sizes proportional to the number of associated individuals. Number of mutations between nodes is represented by slashes unless otherwise noted

Seven genetic indices were estimated from both the cytb and D loop dataset (only U.S. individuals; see Table 4). These included: number of polymorphic sites (*s*) was 66, nucleotide diversity (π) was 0.01618, number of haplotypes (*h*) was 8, haplotype diversity (*H*_d_) was 0.365, and Fu's test of neutrality was 42.287 for the entire cytb dataset (excluding sites with gaps or missing data) and within‐population indices are reported in Table 4 using DNAsp v6 (Rozas et al., 2017). For the entire D loop dataset (excluding sites with gaps or missing data), these included: number of polymorphic sites (*s*) was 116, nucleotide diversity (π) was 0.02296, number of haplotypes (*h*) was 5, haplotype diversity (*H*_d_) was 0.182, and Fu's test of neutrality was 25.498 and within‐population indices are reported in Table 5 using DNAsp v6 (Rozas et al., 2017). Tajima's D for the cytb dataset was 1.97067 and was not significant (.10 > *p* > .05) whereas Tajima's D for the D loop dataset was −1.3532 and was not significant (*p* = 0.068) using the program Arlequin (version 3.5.2.2., Excoffier et al., 1992; Excoffier & Lischer, 2010; Weir, 1996; Weir & Cockerham, 1984). For the D loop dataset, nine haplotypes were identified by the program Network 10.2.0.0 (Bandelt et al., 1999; Fluxus Technology Ltd 2021), which excluded indel events (Table 1), whereas 12 haplotypes were identified for the cytb dataset. The haplotype networks for the cytb (Figure 7) and D loop (Figure 8) datasets placed haplotypes into two major groups (1 and 2), that were similar in content to Clades I and II obtained from the phylogenetic analyses. For the cytb dataset, Group I contained five haplotypes (that differed by 6 total substitutions), Group II‐A contained two haplotypes (that differed by 1 substitution), and Group II‐B contained five haplotypes (that differed by 5 total substitutions). However, Groups I and II differed by 15 total substitutions. For the D loop dataset, Group I contained four haplotypes (that differed by 29 total substitutions), Group II‐A contained 4 haplotypes (that differed by 25 total substitutions), and Group II‐B contained 1 haplotype. However, Groups I and II differed by 90 total substitutions.

**TABLE 4:** The number of polymorphic sites (*s*), nucleotide diversity (π), number of haplotypes (*h*), haplotype diversity (*H*_d_), and Fu's test of neutrality calculated for the entire aoudad cytb dataset (excluding sequences from individuals located outside of the US)

Sampling location	Clade I *n*	Clade II‐A *n*	Clade II‐B *n*	*s*	H	H_d_	π	F*_s_*	Tajima's D (*p* value)
All	46	2	184	66	8	0.365	0.01618	42.287	1.97067 (0.10 > *p* > .05)
Palo Duro Canyon State Park	0	0	14	0	0	0	0	0	0
Caprock Canyons State Park	0	0	7	1	2	0.286	0.00025	−0.095	−1.00623 (*p* > .10)
Garza Co.	1	0	6	57	2	0.286	0.01429	11.308	−1.73872 (**, *p* < .01)
Scurry Co.	0	0	4	0	0	0	0	0	0
Fawcett WMA	9	0	12	59	4	0.61	0.02619	22.862	3.2898 (***, *p* < .001)
Fossil Rim Wildlife Center	19	0	0	1	2	0.199	0.00017	−0.055	−0.56216 (*p* > .10)
Culberson Co.	0	0	1	NA	NA	NA	NA	NA	NA
Carrizo Mountains	0	0	9	0	0	0	0	0	0
Van Horn Mountains	0	0	10	0	0	0	0	0	0
Davis Mountains	1	1	8	61	3	0.378	0.01127	9.417	−1.98573 (**, *p* < .01)
Sierra Viejas Mountains	0	0	10	0	0	0	0	0	0
Chinati Mountains	0	0	28	1	2	0.071	0.00006	−1.155	−1.15142 (*p* > .10)
Elephant Mountain WMA	1	1	3	61	3	0.7	0.02219	6.133	−1.02836 (*p* > .10)
Glass Mountains	3	0	0	0	0	0	0	0	0
Black Gap WMA	3	0	15	57	2	0.294	0.01471	21.41	0.04814 (*p* > .10)
Dolan Falls Preserve	3	0	32	57	2	0.161	0.00807	20.448	−1.22751 (*p* > .10)
Kerr WMA	1	0	1	57	2	1	0.05	4.043	NA
Love Creek Preserve	4	0	7	58	3	0.564	0.02584	15.933	2.31117 (*, *p* < .05)
Val Verde Co.	1	0	0	NA	NA	NA	NA	NA	NA
Presidio Co.	0	0	8	0	0	0	0	0	0
Alamogordo, NM	0	0	3	0	0	0	0	0	0
Lincoln Co., NM (GMU 37)	0	0	3	0	0	0	0	0	0
San Simeon, CA	0	0	3	0	0	0	0	0	0
Clade I	46	0	0	7	4	0.467	0.00072	0.291	−1.30292 (*p* > .10)
Clade II‐A	0	2	0	0	0	0	0	0	0
Clade II‐B	0	0	184	2	3	0.022	0.00002	−4.969	−1.29621 (*p* > .10)

**TABLE 5:** The number of polymorphic sites (*s*), nucleotide diversity (π), number of haplotypes (*h*), haplotype diversity (*H*_d_), and Fu's test of neutrality calculated for the entire aoudad D loop dataset (excluding sequences from individuals located outside of the United States)

Sampling location	Clade I *n*	Clade II‐A *n*	Clade II‐B *n*	*s*	H	H_d_	π	F*_s_*	Tajima's D	*p*value
All	5	1	57	116	5	0.182	0.02296	25.498	−1.30532	.068
Palo Duro Canyon SP	0	0	14	0	0	0	0	0	0	1
Caprock Canyons SP	0	0	2	0	0	0	0	0	0	1
Garza Co.	1	0	0	NA	NA	NA	NA	NA	NA	NA
Fawcett WMA	1	0	4	125	2	0.400	0.04673	12.664	−1.26644	<.001
Culberson Co.	0	0	1	NA	NA	NA	NA	NA	NA	NA
Van Horn Mountains	0	0	9	8	7	0.917	0.00176	−3.892	0	1
Sierra Vieja Mountains	0	0	3	1	2	0.667	0.0006	0.201	0	1
Chinati Mountains	0	0	8	1	2	0.250	0.00028	−0.182	0	1
Elephant Mountain WMA	0	1	0	NA	NA	NA	NA	NA	NA	NA
Dolan Falls Preserve	2	0	10	98	2	0.303	0.04432	21.780	−0.39554	.347
Love Creek Preserve	1	0	2	125	2	0.667	0.07795	8.188	0	1
New Mexico	0	0	1	NA	NA	NA	NA	NA	NA	NA
California	0	0	3	1	2	0.667	0.0006	0.201	0	1
Clade I	5	0	0	21	4	0.900	0.00801	1.395	−0.99781	.181
Clade II	0	1	57	25	2	0.034	.00123	3.522	−2.67714	<.001
Clade II‐A	0	1	0	NA	NA	NA	NA	NA	NA	NA
Clade II‐B	0	0	57	0	0	0	0	0	0	1

### 3.6 Characterization of PRNP exon 3

DNA sequences from exon 3 of the *PRNP* gene were obtained from 10 individual aoudad revealed that all sequences were monomorphic. Translation of nucleotides to amino acids revealed that aoudad possessed the signature genotype of A136, R154, and Q171 (Table 6), which is the most common genotype among sheep and goats (Goldmann, 2008).

**TABLE 6:** Comparative region of the prion protein (PrP) showing aoudad, Texas desert bighorn sheep, and the five most common genotypes in domestic sheep and goats

GenBank Accession Number	Species	PrP Genotype	Portion of PrP Sequence
This study	*Ammotragus lervia*	ARQ	GAVVGGLGGYMLGSAMSRPLIHFGNDYEDRYYRENMYRYPNQVYYRPVDQYSNQNNFVHDC
This study	*Ovis canadensis*	ARQ	GAVVGGLGGYMLGSAMSRPLIHFGNDYEDRYYRENMYRYPNQVYYRPVDQYSNQNNFVHDC
DQ149332	*O. aries*	ARQ	GAVVGGLGGYMLGSAMSRPLIHFGNDYEDRYYRENMYRYPNQVYYRPVDQYSNQNNFVHDC
AY907685	*O. aries*	VRQ	GAVVGGLGGYMLGSVMSRPLIHFGNDYEDRYYRENMYRYPNQVYYRPVDQYSNQNNFVHDC
DQ272610	*O. aries*	ARH	GAVVGVLGGYMLGSAMSRPLIHFGNDYEDRYYRENMYRYPNQVYYRPVDHYSNQNNFVHDC
DQ149333	*O. aries*	ARR	GAVVGGLGGYMLGSAMSRPLIHFGNDYEDRYYRENMYRYPNQVYYRPVDRYSNQNNFVHDC
DQ149351	*O. aries*	AHQ	GAVVGGLGGYMLGSAMSRPLIHFGNDYEDRYYHENMYRYPNQVYYRPVDQYSNQNNFVHDC

## 4 DISCUSSION

It is important to note that data used in this research were obtained from three independent studies: cytb only (Derouiche et al., 2020), D loop only (Stipoljev et al., 2021), and cytb and D loop combined (this study). Further, the cytb dataset generated herein contained 232 individuals, whereas the D loop dataset contained a select subset of those individuals (*n* = 63). Given the similarity of the results of the cytb and D loop sequence analyses from all three studies, inferences from the smaller D loop dataset can be inferred using the larger cytb dataset. The *PRNP* gene provided negligible information relative to phylogenetic association and source‐stock determination; therefore, discussion was restricted solely to the basic description of the prion protein (PrP) genotype in aoudad.

Phylogenetic analyses obtained from sequence data from the two mitochondrial markers (cytb and D loop) produced a similar arrangement of individuals with two major clades identified (I and II; Figures 2 and 3), as well as a subdivision within Clade II (A and B). Clade I was comprised of presumed progeny of individuals resulting from introductions to Texas and Spain as well as individuals sampled from their native range of western and southeastern Algeria and other potential native origins. Individuals comprising Clade II are thought to be the result of progeny of past introductions to California, New Mexico, Texas, and Europe as well as naturally occurring individuals from their native range of northern Algeria and other potential native origins.

Although the topologies obtained from the cytb and D loop analyses essentially were identical, the branch lengths differed as the result of 10 indel events represented by 64 nucleotide substitutions (Table 1) in the D loop dataset. For example, Clade I, of the cytb phylogeny, had shorter branch lengths in comparison to Clade II; whereas Clade I, of the D loop phylogeny, had longer branch lengths than Clade II, which conveyed the appearance of these mitochondrial markers evolving at different rates. Approximately 96% of the indels occurred within the first 325 bp frame (from the 5’ point of origin for D loop) with several being phylogenetically informative. For example, a 15‐bp deletion event was restricted to individuals in Clade II and a separate 16 bp deletion event was restricted to individuals in Clade I. Collectively, these indels contributed to the greater branch lengths depicted in the D loop topology relative to the cytb dataset.

Genetic divergence values between Clades I and II were 5.12% and 13.88% for cytb and D loop, respectively. Genetic distances obtained from the cytb gene (Table 2) indicated that the level of divergence between Clades I and II was much higher compared to values reported for other closely‐related subspecies of bovids ($\bar{x}$ = 1.8%; e.g., *Rupicapra rupicapra*, *Rupicapra pyrenaica*, and *Budorcas taxicolor*, respectively). In addition, the genetic divergences of D loop observed between the two clades of aoudad (I and II) indicated a high level of genetic divergence compared to subspecies of *Rupicapra* ($\bar{x}$ = 8.04%; Table 3). The high levels of genetic divergence detected between Clades I and II indicates a magnitude of genetic divergence typically distinguishing subspecies of mammals (Baker & Bradley, 2006; Bradley & Baker, 2001).

Divergence dating analyses indicated that the *Ammotragus* diverged from *Arabitragus* approximately 3.68 mya, similar to that estimated from more extensive studies examining the timing of divergences of various members of the Cetartiodactyla (Hassanin et al., 2012). This divergence was followed by a radiation of *Ammotragus* into two major clades (I and II) at 2.38 mya. Similar divergence estimates were reported for *Ammotragus* by Derouiche et al. (2020). Radiation within *A. l. sahariensis* (Clade I) was estimated to have occurred at 0.99 mya. The major split between individuals assigned to Subclade II‐A and Subclade II‐B was estimated to have occurred within the last 1.25 mya. Radiations within Subclade II‐A and Subclade II‐B were estimated at 0.65 and 0.85 mya, respectively. The divergence between Clades I and II and subsequent radiations within each clade were similar to that estimated for recognized subspecies of bovids (*Bos*, *Capra*, *Kobus*, *Redunca*, *Rupicapra*, *Tragelaphus*; *Capricornis*, and *Naemorhedus*; Derouiche et al., 2020; Hassanin et al., 2012) demonstrating the significant genetic divergence existing between Clades I and II.

Variable sites in the cytb dataset ranged from zero between individuals located within clades to a maximum of 66 between Clades I and II. Similar haplotype and nucleotide diversity values compared to Derouiche et al. (2020) were obtained in some populations, specifically Fawcett WMA and Love Creek Preserve (Table 4). Variable sites in the D loop dataset ranged from zero between individuals located within clades to a maximum of 125 between Clades I and II. Haplotype and nucleotide diversity values obtained from Texas populations (Table 5) were similar to those obtained from European populations reported in Stipoljev et al. (2021). The haplotype networks generated in the cytb and D loop datasets (Figures 4 and 5) indicated a separation of haplotypes into two groups that were similar in composition to each other and to the Clades obtained in the phylogenetic analyses. For the cytb dataset, four populations were identified by a test of Tajima's D as statistically significant (Table 4). Two of these populations (Garza County and Davis Mountains) were characterized by a negative Tajima's D, which may be interpreted that these populations recently were under a selective sweep characterized by population expansion (Tajima, 1989). In contrast, Fawcett WMA and Love Creek were identified by a positive Tajima's D, which may be indicative of balancing selection and population contraction (Tajima, 1989). For the D loop dataset, two populations (Fawcett WMA and Clade II as a whole) were identified by a test of Tajima's D as statistically significant (Table 5). The negative values of Tajima's D for these two populations may be interpreted as population expansion as a result of a recent selective sweep (Tajima, 1989).

Based on the phylogenetic and genetic divergences, below we provide an interpretation for the origin of populations in the United States and Europe. Concerning aoudad introductions to United States, phylogenetic support and genetic divergences values indicated the presence of three haplogroups. The most abundant and widely distributed (California, New Mexico, and Texas) haplogroup was represented by individuals in Subclade II‐B. The second most common haplogroup represented by individuals in Clade I was restricted to populations in central and west‐central Texas. The third haplogroup (Subclade II‐A) was at a much lower frequency and was restricted to two localities in extreme western Texas.

Based on the available information garnered from translocation records, state agencies, and scientific publications (Barrett, 1980; Mungall & Sheffield, 1994; Ogren, 1959, 1965; Simpson & Krysl, 1981), it appears that the first translocated aoudad (most likely *A. l. lervia*, Cassinello, 1998; Gray, 1985; Ogren, 1965) in the United States initially were imported from European zoos to zoological parks and ultimately to private ranches. Sources indicated that the first free‐ranging population in the United States was established on the Hearst Ranch (San Lucia Range, California) circa 1925 (Barrett, 1980) most likely sourced from the Fleishhacker Zoo (now the San Francisco Zoo; Mungall & Sheffield, 1994); unfortunately, importation records and other forms of documentation were unavailable and consequently were not useful in establishing the country of origin. Escapees from the Hearst Ranch were thought to have established the contemporary population that currently is restricted to the San Lucia Range. Other individuals from the Hearst Ranch were used to establish zoo populations in California (San Francisco Zoo, Barrett, 1980; San Diego Zoo, Mungall & Sheffield, 1994). In addition, a small number of Hearst Ranch individuals were used by McKnight and Louis Goebal used to establish populations in southeastern (near Picacho) and northwestern (Canyon Largo near Farmington) New Mexico in 1940 and 1956, respectively (Morrison, 1980). Escapees from the McKnight Ranch are thought to have been responsible for establishing free‐ranging populations near Alamogordo and the Hondo Valley, New Mexico, and Guadalupe Mountains near the Texas/New Mexico border (Morrison, 1980). In addition, descendants from the Hearst and McKnight Ranches were used by New Mexico Department of Game and Fish and Texas Parks and Wildlife Department to establish populations in Canadian River Gorge in 1950 (Morrison, 1980) and Palo Duro Canyon in 1957 and 1958 (DeArment, 1971; Mungall & Sheffield, 1994). In fact, the genetic data presented herein suggest that the most common haplogroup (Clade II‐B) that is distributed throughout California, New Mexico, and Texas appears to be a product of descendants from the Hearst and McKnight Ranches.

In an attempt to assign subspecific designation to the original source‐stock events, DNA sequences generated herein were compared to those presented in Derouiche et al. (2020) and Stipoljev et al. (2021). First, it appears that the widespread US haplogroup (Subclade II‐B) is identical to sequences reported for *Ammotragus lervia lervia*. Second, although there are no translocation records (Simpson & Krysl, 1981) for the remaining two haplogroups restricted to Texas, the second‐most common haplogroup (Clade I) appears to be representative of *A. l. sahariensis* or *A. l. blainei*. The difficulty in assigning subspecific origin (see Figure 3) to these samples stems from the fact that genetic data presented in Derouiche et al. (2020) seem to suggest that the Texas samples should be representative of *A. l. sahariensis*. The pedigree data obtained from the Fossil Rim Wildlife Center (FRWC) studbook indicated that individuals from their facility were obtained from the Khartoum Zoo, Sudan circa 1991 and therefore were most likely representative of the Kordofan subspecies, *A. l. blainei* (M. Shea, FRWC, personal communication). However, no samples clearly assignable to *A. l. blainei* were available for this study; therefore, the source‐stock at the Khartoum Zoo were not actually representative of *A. l. blainei* or that *A. l. sahariensis* and *A. l. blainei* are genetically identical based on the mtDNA dataset (this study; Derouiche et al., 2020; Stipoljev et al., 2021). Further, Alados et al. (1988) and Castelló (2016) propose a wider distribution for *A. l. blainei* in the Ennedi and Uweinat mountains in northeast Chad, the native range of this subspecies currently is estimated to occur solely in the Red Hills of east Sudan (Cassinello, 2013; Nimir, 1997). If *A. l. sahariensis* was once widespread in Chad as proposed by Cassinello (2013), it may be that *A. l. sahariensis* and *A. l. blainei* may not represent distinct subspecies. Third, at this time, we cannot definitively determine the affiliation of the rare haplogroup (Subclade II‐A); however, it most likely is either affiliated with *A. l. lervia* or one of the other subspecies that have yet to be genetically examined. Clearly, Clades I and II differ substantially in regard to levels of genetic divergence (see Baker & Bradley, 2006 and Bradley & Baker, 2001 for a discussion) lending credence to the observation that the two clades represent two subspecies and genetically may be sufficiently distinct to be considered different species. Although little morphological variation separates the various subspecies of *A. lervia*, the extreme genetic divergence precludes a closer examination of the taxonomy of this taxon as it relates to the haplogroups.

Relative to Europe, Stipoljev et al. (2021) postulated that, based on translocation records (see Cassinello, 1995), the population from Almería, Spain, (Haplotype Amle02) was representative of the Saharan subspecies (*A. l. sahariensis*). However, when included with the D loop dataset herein, Haplotype Amle02 as well as Haplotype Amle03 grouped with samples from Algeria (*A. l. lervia* or one of the other subspecies that have yet to be genetically examined) contained in Clade II‐A. Additionally, Haplotype Amle01 was associated with Clade II‐B. This discrepancy suggests that Haplotype Amle04 (Sierra Espuña, Spain) most likely is representative of *A. l. sahariensis* as this haplotype groups with Clade I; whereas Haplotypes Amle01, Amle02, and Amle03 and other samples from Texas, are representative of *A. l. lervia* or one of the other subspecies that have yet to be genetically examined. These results imply that additional populations from Almería and surrounding regions should be examined to determine whether they are *A. l. lervia* or *A. l. sahariensis* or if they represent an undescribed genetic subspecies.

## 5 CONCLUSION

### 5.1 Concerns surrounding competition and extinction of sympatric haplogroups

Given the high level of genetic divergence (two major haplogroups ‐ Clades I and II, Figures 2 and 3), it may be prudent to monitor populations to prevent homogenization of subspecies and loss of genetic and morphologic variation in light of their conservation status in their native range. First, it is noteworthy, that in Texas, the two major haplogroups (Clade I and Subclade II‐B) were sympatric at six localities (Figure 3: 3, 5, 15, 16, 17, and 18). At these sites, haplogroup II‐B was always present at a greater frequency, ranging from 57% to 91% (see Table 4). Further, two other localities (Figure 3: 10 and 13) possessed all three haplogroups (Clade I and Subclades II‐A and II‐B) with the following haplogroup composition: Locality 10 – 10%, 10%, and 80% and Locality 13 – 20%, 20%, and 60%. Second, two localities, Fawcett WMA (previously the Waddell Ranch) and Garza County, provided data for a historical and contemporary comparison of change in haplogroup frequency over time. For example, individuals harvested in 1985 from the Waddell Ranch indicated the presence of two haplogroups, Clade I (*n* = 2) and Subclade II‐B (*n* = 1), respectively. Similarly, samples collected at this locality in 2020 indicated that both of these haplogroups persisted over the 40‐year interval (Clade I, *n* = 7 and Subclade II‐B, *n* = 11; respectively). Third, the Dolan Falls Preserve population is highly skewed toward one haplogroup as only three individuals represented Clade I and 32 individuals were assigned to Subclade II‐B. Under this disproportional representation of haplotypes, over time, the haplogroup associated with Subclade II‐B may outcompete the haplogroup associated with Clade I, resulting in a local extinction of the second most common haplotype in United States. Fourth, if haplogroups are indicative of subspecies (see Baker & Bradley, 2006 and Bradley & Baker, 2001 for a discussion), then populations of aoudad in Texas may be comprised of three different subspecies (*A. l. blainei*, *A. l. lervia*, *and A. l. sahariensis*).

On another note, aoudad exist in sympatry with native and charismatic Texas desert bighorn sheep in the Trans‐Pecos Region and therefore the possibility for interspecies competition and disease transmission present concerns to the long‐term management of these species (Barrett, 1967; Seegmiller & Simpson, 1979; Simpson & Krysl, 1981; Simpson et al., 1978). For example, throughout much of Texas and the desert southwest, water is a limited resource and results in the congregation of both species at natural and man‐made water sources. Similarly, both species consume similar forage (Seegmiller & Simpson, 1979; Simpson et al., 1978) and may compete for this resource as well. Given the disparity in population size (>30,000 aoudad to 1500 bighorn sheep; F. Hernández, TPWD, personal communication), free‐ranging populations of aoudad in the Trans‐Pecos region will continue to increase as a result of year‐round reproduction, high levels of recruitment, access to quality habitat and water resources, and minimal hunting pressure; thereby, outcompeting native bighorn sheep and possibly contribute to their extirpation (Barrett, 1967; Seegmiller & Simpson, 1979; Simpson & Krysl, 1981; Simpson et al., 1978). Further, the prion genotype, which confers average susceptibility to diseases such as scrapie (Goldmann, 2008), was detected in aoudad individuals examined in this study (Table 6). Although the risk of prion transmission may be low, bighorn sheep and other native ungulates (e.g., mule deer, white‐tailed deer, elk, and others) in this region may be at risk. For these and other reasons, population control of aoudad may become necessary to counter competition and disease transmission.

## CONFLICT OF INTEREST

The authors declare no conflicts of interest.

## AUTHOR CONTRIBUTION

**Emily Alyse Wright:**Conceptualization (lead); Data curation (lead); Formal analysis (lead); Investigation (lead); Methodology (lead); Project administration (lead); Resources (equal); Validation (equal); Visualization (equal); Writing – original draft (lead); Writing – review & editing (lead). **Rachael C. Wiedmeier:** Data curation (supporting); Investigation (supporting); Resources (supporting); Writing  – review & editing (supporting). **Emma K. Roberts:** Data curation (supporting); Investigation (supporting); Methodology (supporting); Writing – review & editing (supporting). **David R. Pipkin:** Data curation (supporting); Resources (supporting); Writing – review & editing (supporting). **Froylán Hernández:** Data curation (supporting); Resources (supporting); Writing – review & editing (supporting). **Joseph P. Bayouth:** Data curation (supporting); Formal analysis (supporting); Writing – review & editing (supporting). **Warren C. Conway:** Conceptualization (equal); Data curation (supporting); Funding acquisition (lead); Project administration (equal); Resources (equal); Supervision (equal); Validation (equal); Visualization (equal); Writing – review & editing (equal). **Robert D Bradley:** Conceptualization (equal); Data curation (equal); Formal analysis (equal); Funding acquisition (equal); Investigation (equal); Methodology (equal); Project administration (equal); Resources (equal); Software (equal); Supervision (lead); Validation (equal); Visualization (lead); Writing – original draft (equal); Writing – review & editing (equal).

## Supporting information

Supplementary Material

## Data Availability

DNA sequences: NCBI GenBank accession numbers: MZ507707‐MZ507938(cytb); MZ507939‐MZ508001(D loop); MZ508002‐MZ508011(*PRNP*).
