# Supplementary material for: Distinct mtDNA lineages in free‐ranging Ammotragus(aoudad) from the United States indicate multiple introductions from northern Africa
Source: Ecol Evol. 2022 Apr 19;12(4):e8849. doi: 10.1002/ece3.8849 (PMC9018999; doi:10.1002/ece3.8849)
Supplement: Supplementary file 1 — Supplementary Material [file ECE3-12-e8849-s001.docx]

**Appendix**

Specimens examined in this study. For each collected specimen, locality data cross-referenced to Figure 3, haplotype corresponding to Figures 7 and 8 (cytochrome-*b* and D-loop, respectively), museum catalog number (abbreviations for museum acronyms follow Hafner et al. 1997), and GenBank accession number (cytochrome-*b*, D-loop, and exon 3 of the prion protein gene, respectively) are provided in parentheses. Abbreviations are as follows: Angelo State Natural History Collection (ASNHC) and Museum of Texas Tech University (TTU). If museum catalog numbers were unavailable, specimens were referenced with a corresponding special number (e.g., ASK special number of the Angelo State Natural History Collection; color codes for Fossil Rim Wildlife Center; and TK number special number of the Museum of Texas Tech University).

*Ammotragus lervia*.---UNITED STATES: California; San Luis Obispo County, 5 mi E Cambria (Locality 23, Haplotype 9, Haplotype 9, TK259888, MZ507807, MZ507992, NA; Locality 23, Haplotype 9, Haplotype 9, TK259889, MZ507808, MZ507993, NA; Locality 23, Haplotype 9, Haplotype 9, TK259890, MZ507809, MZ507994, NA).

*Ammotragus lervia*.---UNITED STATES: New Mexico; Otero County, Game Management Unit (GMU) 34, 32.63707, -105.83423 (Locality 21, Haplotype 9, Haplotype 9, TK259882, MZ507801, MZ507990, NA; Locality 21, Haplotype 9, NA, TK259883, MZ507802, NA, NA; Locality 21, Haplotype 9, NA, TK259887, MZ507806, NA, NA); Lincoln County, GMU 37 (Locality 22, Haplotype 9, NA, TK259972, MZ507838, NA, NA; Locality 22, Haplotype 9, NA, TK259977, MZ507843, NA, NA; Locality 22, Haplotype 9, NA, TK259979, MZ507845, NA, NA).

*Ammotragus lervia*.---UNITED STATES: Texas; Bandera County, Love Creek Preserve (Locality 20, Haplotype 3, Haplotype 3, TK259870, MZ507794, MZ507984, MZ508008; Locality 20, Haplotype 9, Haplotype 9, TK259871, MZ507795, MZ507985, NA; Locality 20, Haplotype 9, Haplotype 9, TK259872, MZ507796, MZ507986, NA; Locality 20, Haplotype 9, NA, TK259932, MZ507827, NA, NA; Locality 20, Haplotype 4, NA, TK259933, MZ507828, NA, NA; Locality 20, Haplotype 4, NA, TK259934, MZ507829, NA, NA; Locality 20, Haplotype 4, NA, TK259935, MZ507830, NA, NA; Locality 20, Haplotype 9, NA, TK259936, MZ507831, NA, NA; Locality 20, Haplotype 9, NA, TK259937, MZ507832, NA, NA; Locality 20, Haplotype 9, NA, TK259967, MZ507833, NA, NA; Locality 20, Haplotype 9, NA, TK259968, MZ507834, NA, NA).

*Ammotragus lervia*.---UNITED STATES: Texas; Brewster County, Glass Mountains (Locality 15, Haplotype 3, NA, TK259998, MZ507864, NA, NA; Locality 15, Haplotype 3, NA, TK259999, MZ507865, NA, NA; Locality 15, Haplotype 3, NA, TK260000, MZ507866, NA, NA), Elephant Mountain Wildlife Management Area (WMA) (Locality 14, Haplotype 7, Haplotype 8, TK260026, MZ507892, MZ508000, NA; Locality 14, Haplotype 9, NA, TK260027, MZ507893, NA, NA; Locality 14, Haplotype 9, NA, TK260028, MZ507894, NA, NA; Locality 14, Haplotype 9, NA, TK260029, MZ507895, NA, NA; Locality 14, Haplotype 2, NA, TK260030, MZ507896, NA, NA); Black Gap WMA (Locality 16, Haplotype 9, NA, TK260031, MZ507897, NA, NA; Locality 16, Haplotype 9, NA, TK260032, MZ507898, NA, NA; Locality 16, Haplotype 9, NA, TK260033, MZ507899, NA, NA; Locality 16, Haplotype 9, NA, TK260034, MZ507900, NA, NA; Locality 16, Haplotype 9, NA, TK260035, MZ507901, NA, NA; Locality 16, Haplotype 9, NA, TK260036, MZ507902, NA, NA; Locality 16, Haplotype 9, NA, TK260037, MZ507903, NA, NA; Locality 16, Haplotype 9, NA, TK260038, MZ507904, NA, NA; Locality 16, Haplotype 3, NA, TK260039, MZ507905, NA, NA; Locality 16, Haplotype 3, NA, TK260040, MZ507906, NA, NA; Locality 16, Haplotype 9, NA, TK260041, MZ507907, NA, NA; Locality 16, Haplotype 9, NA, TK260042, MZ507908, NA, NA; Locality 16, Haplotype 9, NA, TK260043, MZ507909, NA, NA; Locality 16, Haplotype 9, NA, TK260044, MZ507910, NA, NA; Locality 16, Haplotype 9, NA, TK260045, MZ507911, NA, NA; Locality 16, Haplotype 9, NA, TK260046, MZ507912, NA, NA; Locality 16, Haplotype 9, NA, TK260047, MZ507913, NA, NA; Locality 16, Haplotype 3, NA, TK260048, MZ507914, NA, NA).

*Ammotragus lervia*.---UNITED STATES: Texas; Briscoe County, Caprock Canyons State Park (Locality 2, Haplotype 9, Haplotype 9, TK256667, MZ507748, MZ507970, MZ508004; (Locality 2, Haplotype 9, Haplotype 9, TK256668, MZ507749, MZ507971, MZ508005; Locality 2, Haplotype 9, NA, TK259899, MZ507818, NA, NA; Locality 2, Haplotype 9, NA, TK259900, MZ507819, NA, NA; Locality 2, Haplotype 9, NA, TK259901, MZ507820, NA, NA; Locality 2, Haplotype 9, NA, TK259902, MZ507821, NA, NA; Locality 2, Haplotype 8, NA, TK259903, MZ507822, NA, NA).

*Ammotragus lervia*.---UNITED STATES: Texas; Culberson County, Van Horn Mountains, 12 mi S Van Horn US-90 (Locality 10, Haplotype 9, Haplotype 9, TK249427, MZ507707, MZ507939, NA; Locality 10, Haplotype 9, Haplotype 9, TK249428, MZ507708, MZ507940, NA; Locality 10, Haplotype 9, Haplotype 9, TK249429, MZ507709, MZ507941, NA; Locality 10, Haplotype 9, Haplotype 9, TK249430, MZ507710, MZ507942, NA; Locality 10, Haplotype 9, Haplotype 9, TK249431, MZ507711, MZ507943, NA; Locality 10, Haplotype 9, Haplotype 9, TK249432, MZ507712, MZ507944, NA; Locality 10, Haplotype 9, Haplotype 9, TK249433, MZ507713, MZ507945, NA; Locality 10, Haplotype 9, Haplotype 9, TK249434, MZ507714, NA, NA); Patterson Hills (Locality 7, Haplotype 9, Haplotype 9, ASK14306, MZ507915, MZ508001, NA).

*Ammotragus lervia*.---UNITED STATES: Texas; Garza County, near Post (Locality 3, Haplotype 9, NA, TK259873, MZ507797, NA, MZ508009; Locality 3, Haplotype 9, NA, TK259893, MZ507812, NA, NA; Locality 3, Haplotype 9, NA, TK260018, MZ507884, NA, NA; Locality 3, Haplotype 9, NA, TTU-M152093, MZ507919, NA, NA); W HWY 84 (Locality 3, Haplotype 3, Haplotype 3, TK259884, MZ507803, MZ507991, MZ508010); E HWY 84 (Locality 3, Haplotype 9, NA, TK259885, MZ507804, NA, NA); 12.5 mi SE Post (Locality 3, Haplotype 9, NA, TTU-M152094, MZ507823, NA, NA; Locality 3, Haplotype 9, NA, TK259929, MZ507824, NA, NA).

*Ammotragus lervia*.---UNITED STATES: Texas; Hudspeth County, Van Horn Mountains (Locality 10, Haplotype 9, Haplotype 9, TK249435, MZ507715, MZ507946, NA; Locality 10, Haplotype 9, Haplotype 9, TK249436, MZ507716, MZ507947, NA); Carrizo Mountains (Locality 9, Haplotype 9, NA, TK259802, MZ507772, NA, NA; Locality 9, Haplotype 9, NA, TK259803, MZ507773, NA, NA; Locality 9, Haplotype 9, NA, TK259804, MZ507774, NA, NA; Locality 9, Haplotype 9, NA, TK259805, MZ507775, NA, NA; Locality 9, Haplotype 9, NA, TK259806, MZ507776, NA, NA; Locality 9, Haplotype 9, NA, TK259807, MZ507777, NA, NA; Locality 9, Haplotype 9, NA, TK259808, MZ507778, NA, NA; Locality 9, Haplotype 9, NA, TK259809, MZ507779, NA, NA; Locality 9, Haplotype 9, NA, TK259810, MZ507780, NA, NA).

*Ammotragus lervia*.---UNITED STATES: Texas; Jeff Davis County, Davis Mountains, 30.88917, -104.24139 (Locality 8, Haplotype 9, NA, TK259981, MZ507847, NA, NA; Locality 8, Haplotype 9, NA, TK259982, MZ507848, NA, NA; Locality 8, Haplotype 9, NA, TK259983, MZ507849, NA, NA; Locality 8, Haplotype 9, NA, TK259984, MZ507850, NA, NA); southern portion of Davis Mountains (Locality 8, Haplotype 9, NA, TK260022, MZ507888, NA, NA; Locality 8, Haplotype 7, NA, TK260023, MZ507889, NA, NA; Locality 8, Haplotype 9, NA, TK260024, MZ507890, NA, NA; Locality 8, Haplotype 3, NA, TK260025, MZ507891, NA, NA).

*Ammotragus lervia*.---UNITED STATES: Texas; Kerr County, Kerr WMA (Locality 19, Haplotype 9, NA, TK259930, MZ507825, NA, NA; Locality 19, Haplotype 2, NA, TK259931, MZ507826, NA, NA).

*Ammotragus lervia*.---UNITED STATES: Texas; Palo Pinto County, Fawcett WMA (Locality 5, Haplotype 9, Haplotype 9, TK259894, MZ507813, MZ507995, NA; Locality 5, Haplotype 9, Haplotype 9, TK259895, MZ507814, MZ507996, NA; Locality 5, Haplotype 9, Haplotype 9, TK259896, MZ507815, MZ507997, NA; Locality 5, Haplotype 9, Haplotype 9, TK259897, MZ507816, MZ507998, NA; Locality 5, Haplotype 4, Haplotype 4, TK259898, MZ507817, MZ507999, NA; Locality 5, Haplotype 4, NA, TK259985, MZ507851, NA, NA; Locality 5, Haplotype 9, NA, TK259986, MZ507852, NA, NA; Locality 5, Haplotype 4, NA, TK259987, MZ507853, NA, NA; Locality 5, Haplotype 5, NA, TK259988, MZ507854, NA, NA; Locality 5, Haplotype 9, NA, TK259989, MZ507855, NA, NA; Locality 5, Haplotype 9, NA, TK259990, MZ507856, NA, NA; Locality 5, Haplotype 9, NA, TK259991, MZ507857, NA, NA; Locality 5, Haplotype 4, NA, TK259992, MZ507858, NA, NA; Locality 5, Haplotype 9, NA, TK259993, MZ507859, NA, NA; Locality 5, Haplotype 4, NA, TK259994, MZ507860, NA, NA; Locality 5, Haplotype 4, NA, TK259995, MZ507861, NA, NA; Locality 5, Haplotype 9, NA, TK259996, MZ507862, NA, NA; Locality 5, Haplotype 9, NA, TK259997, MZ507863, NA, NA); Waddell Ranch (now Fawcett WMA) (Locality 5, Haplotype 3, NA, TK174854, MZ507916, NA, NA; Locality 5, Haplotype 9, NA, TK174855, MZ507917, NA, NA; Locality 5, Haplotype 3, NA, TK174856, MZ507918, NA, NA).

*Ammotragus lervia*.---UNITED STATES: Texas; Presidio County (Locality 12, Haplotype 9, NA, TK259969, MZ507835, NA, NA; Locality 12, Haplotype 9, NA, TK259970, MZ507836, NA, NA; Locality 12, Haplotype 9, NA, TK259971, MZ507837, NA, NA; Locality 12, Haplotype 9, NA, TK259973, MZ507839, NA, NA; Locality 12, Haplotype 9, NA, TK259974, MZ507840, NA, NA; Locality 12, Haplotype 9, NA, TK259976, MZ507842, NA, NA; Locality 12, Haplotype 9, NA, TK259978, MZ507844, NA, NA; Locality 12, Haplotype 9, NA, TK259980, MZ507846, NA, NA); Sierra Viejas (Locality 11, Haplotype 9, Haplotype 9, TK249437, MZ507717, MZ507948, NA; Locality 11, Haplotype 9, Haplotype 9, TK249438, MZ507718, MZ507949, NA; Locality 11, Haplotype 9, Haplotype 9, TK249439, MZ507719, MZ507950, NA; Locality 11, Haplotype 9, NA, TK249440, MZ507720, NA, NA; Locality 11, Haplotype 9, NA, TK249441, MZ507721, NA, NA; Locality 11, Haplotype 9, NA, TK249442, MZ507722, NA, NA; Locality 11, Haplotype 9, NA, TK249443, MZ507723, NA, NA; Locality 11, Haplotype 9, NA, TK249444, MZ507724, NA, NA; Locality 11, Haplotype 9, NA, TK249445, MZ507725, NA, NA; Locality 11, Haplotype 9, NA, TK256511, MZ507726, NA, NA); Chinati Mountains (Locality 13, Haplotype 9, Haplotype 9, TK256526, MZ507727, MZ507951, NA; Locality 13, Haplotype 9, Haplotype 9, TK256527, MZ507728, MZ507952, NA; Locality 13, Haplotype 9, Haplotype 9, TK256528, MZ507729, MZ507953, NA; Locality 13, Haplotype 9, Haplotype 9, TK256529, MZ507730, MZ507954, NA; Locality 13, Haplotype 9, Haplotype 9, TK256531, MZ507731, MZ507955, NA; Locality 13, Haplotype 9, Haplotype 9, TK256532, MZ507732, MZ507956, NA; Locality 13, Haplotype 9, Haplotype 9, TK256533, MZ507733, MZ507957, NA; Locality 13, Haplotype 9, Haplotype 9, TK256534, MZ507734, MZ507958, NA; Locality 13, Haplotype 9, NA, TK256535, MZ507735, NA, NA; Locality 13, Haplotype 9, NA, TK256536, MZ507736, NA, NA; Locality 13, Haplotype 9, NA, TK260008, MZ507874, NA, NA; Locality 13, Haplotype 9, NA, TK260009, MZ507875, NA, NA; Locality 13, Haplotype 9, NA, TK260010, MZ507876, NA, NA; Locality 13, Haplotype 9, NA, TK260011, MZ507877, NA, NA; Locality 13, Haplotype 9, NA, TK260012, MZ507878, NA, NA; Locality 13, Haplotype 9, NA, TK260013, MZ507879, NA, NA; Locality 13, Haplotype 9, NA, TK260014, MZ507880, NA, NA; Locality 13, Haplotype 9, NA, TK260015, MZ507881, NA, NA; Locality 13, Haplotype 9, NA, TK260016, MZ507882, NA, NA; Locality 13, Haplotype 9, NA, TK260017, MZ507883, NA, NA; Locality 13, Haplotype 9, NA, TK260019, MZ507885, NA, NA; Locality 13, Haplotype 9, NA, TK260020, MZ507886, NA, NA; Locality 13, Haplotype 9, NA, TK260021, MZ507887, NA, NA); 35 mi SW Marfa (Locality 13, Haplotype 9, NA, TK260003, MZ507869, NA, NA; Locality 13, Haplotype 9, NA, TK260004, MZ507870, NA, NA; Locality 13, Haplotype 9, NA, TK260005, MZ507871, NA, NA; Locality 13, Haplotype 9, NA, TK260006, MZ507872, NA, NA; Locality 13, Haplotype 9, NA, TK260007, MZ507873, NA, NA)

*Ammotragus lervia*.---UNITED STATES: Texas; Randall County, Palo Duro Canyon State Park (Locality 1, Haplotype 9, Haplotype 9, TK256656, MZ507737, MZ507959, NA; Locality 1, Haplotype 9, Haplotype 9, TK256657, MZ507738, MZ507960, NA; Locality 1, Haplotype 9, Haplotype 9, TK256658, MZ507739, MZ507961, NA; Locality 1, Haplotype 9, Haplotype 9, TK256659, MZ507740, MZ507962, NA; Locality 1, Haplotype 9, Haplotype 9, TK256660, MZ507741, MZ507963, NA; Locality 1, Haplotype 9, Haplotype 9, TK256661, MZ507742, MZ507964, NA; Locality 1, Haplotype 9, Haplotype 9, TK256662, MZ507743, MZ507965, NA; Locality 1, Haplotype 9, Haplotype 9, TK256663, MZ507744, MZ507966, NA; Locality 1, Haplotype 9, Haplotype 9, TK256664, MZ507745, MZ507967, NA; Locality 1, Haplotype 9, Haplotype 9, TK256665, MZ507746, MZ507968, MZ508002; Locality 1, Haplotype 9, Haplotype 9, TK256666, MZ507747, MZ507969, NA; Locality 1, Haplotype 9, Haplotype 9, TK259874, MZ507798, MZ507987, NA; Locality 1, Haplotype 9, Haplotype 9, TK259875, MZ507799, MZ507988, NA; Locality 1, Haplotype 9, Haplotype 9, TK259876, MZ507800, MZ507989, NA).

*Ammotragus lervia*.---UNITED STATES: Texas; Reeves County, Davis Mountains (Locality 8, Haplotype 9, NA, TK260001, MZ507867, NA, NA; Locality 8, Haplotype 9, NA, TK260002, MZ507868, NA, NA).

*Ammotragus lervia*.---UNITED STATES: Texas; Scurry County, near Fluvanna (Locality 4, Haplotype 9, NA, TK259886, MZ507805, NA, NA; Locality 4, Haplotype 9, NA, TK259891, MZ507810, NA, NA; Locality 4, Haplotype 9, NA, TK259892, MZ507811, NA, NA).

*Ammotragus lervia*.---UNITED STATES: Texas; Somervell County, Fossil Rim Wildlife Center (Locality 6, Haplotype 3, NA, 648 White, MZ507920, NA, NA; Locality 6, Haplotype 3, NA, 650 White, MZ507921, NA, NA; Locality 6, Haplotype 4, NA, 695 Yellow, MZ507922, NA, NA; Locality 6, Haplotype 3, NA, 831 Yellow, MZ507923, NA, NA; Locality 6, Haplotype 3, NA, 802 Orange, MZ507924, NA, NA; Locality 6, Haplotype 3, NA, 797 Red, MZ507925, NA, NA; Locality 6, Haplotype 3, NA, 785 Blue, MZ507926, NA, NA; Locality 6, Haplotype 3, NA, 779 Orange, MZ507927, NA, NA; Locality 6, Haplotype 3, NA, 777 Orange, MZ507928, NA, NA; Locality 6, Haplotype 3, NA, 763 Yellow, MZ507929, NA, NA; Locality 6, Haplotype 3, NA, 763 White, MZ507930, NA, NA; Locality 6, Haplotype 3, NA, 763 Blue, MZ507931, NA, NA; Locality 6, Haplotype 3, NA, 755 White, MZ507932, NA, NA; Locality 6, Haplotype 3, NA, 745 Orange, MZ507933, NA, NA; Locality 6, Haplotype 4, NA, 730 Yellow, MZ507934, NA, NA; Locality 6, Haplotype 3, NA, 707 White, MZ507935, NA, NA; Locality 6, Haplotype 3, NA, 790 Orange, MZ507936, NA, NA; Locality 6, Haplotype 3, NA, 826 Red, MZ507937, NA, NA; Locality 6, Haplotype 3, NA, 654 White, MZ507938, NA, NA).

*Ammotragus lervia*.---UNITED STATES: Texas; Val Verde County (Locality 17, Haplotype 3, NA, TK259975, MZ507841, NA, NA); Dolan Falls Preserve (Locality 18, Haplotype 9, NA, TK259778, MZ507750, NA, NA; Locality 18, Haplotype 9, NA, TK259779, MZ507751, NA, NA; Locality 18, Haplotype 3, NA, TK259780, MZ507752, NA, NA; Locality 18, Haplotype 9, NA, TK259781, MZ507753, NA, NA; Locality 18, Haplotype 9, NA, TK259782, MZ507754, NA, NA; Locality 18, Haplotype 9, NA, TK259783, MZ507755, NA, NA; Locality 18, Haplotype 9, NA, TK259784, MZ507756, NA, NA; Locality 18, Haplotype 9, NA, TK259785, MZ507757, NA, NA; Locality 18, Haplotype 9, NA, TK259786, MZ507758, NA, NA; Locality 18, Haplotype 9, NA, TK259787, MZ507759, NA, NA; Locality 18, Haplotype 9, NA, TK259788, MZ507760, NA, NA; Locality 18, Haplotype 9, NA, TK259789, MZ507761, NA, NA; Locality 18, Haplotype 9, NA, TK259790, MZ507762, NA, NA; Locality 18, Haplotype 9, NA, TK259791, MZ507763, NA, NA; Locality 18, Haplotype 9, NA, TK259792, MZ507764, NA, NA; Locality 18, Haplotype 9, NA, TK259793, MZ507765, NA, NA; Locality 18, Haplotype 9, NA, TK259794, MZ507766, NA, NA; Locality 18, Haplotype 9, NA, TK259795, MZ507767, NA, NA; Locality 18, Haplotype 9, NA, TK259796, MZ507768, NA, NA; Locality 18, Haplotype 9, NA, TK259797, MZ507769, NA, NA; Locality 18, Haplotype 9, NA, TK259798, MZ507770, NA, NA; Locality 18, Haplotype 9, NA, TK259799, MZ507771, NA, NA; Locality 18, Haplotype 9, NA, TK259844, MZ507781, NA, NA; Locality 18, Haplotype 3, Haplotype 3, TK259857, MZ507782, MZ507972, MZ508006; Locality 18, Haplotype 3, Haplotype 3, TK259859, MZ507783, MZ507973, MZ508007; Locality 18, Haplotype 9, Haplotype 9, TK259860, MZ507784, MZ507974, NA; Locality 18, Haplotype 9, Haplotype 9, TK259861, MZ507785, MZ507975, NA; Locality 18, Haplotype 9, Haplotype 9, TK259862, MZ507786, MZ507976, NA; Locality 18, Haplotype 9, Haplotype 9, TK259863, MZ507787, MZ507977, NA; Locality 18, Haplotype 9, Haplotype 9, TK259864, MZ507788, MZ507978, NA; Locality 18, Haplotype 9, Haplotype 9, TK259865, MZ507789, MZ507979, NA; Locality 18, Haplotype 9, Haplotype 9, TK259866, MZ507790, MZ507980, NA; Locality 18, Haplotype 9, Haplotype 9, TK259867, MZ507791, MZ507981, NA; Locality 18, Haplotype 9, Haplotype 9, TK259868, MZ507792, MZ507982, NA; Locality 18, Haplotype 9, Haplotype 9, TK259869, MZ507793, MZ507983, NA).
